# Reversal of filarial serpin Wb123-urokinase plasminogen activator receptor mediated alternative macrophage activation by monoclonal antibody

**DOI:** 10.1371/journal.pntd.0013726

**Published:** 2025-12-22

**Authors:** Prince Upadhyay, Akshay Munjal, Abir Mondal, Gagandeep Singh, Soumyadeep Mukherjee, Shagun Siwach, Millee Chandoulla, Mahesh C. Kaushik, Puneet K. Gupta, Soumya Pati, Shailja Singh

**Affiliations:** 1 Department of Life Sciences, School of Natural Sciences, Shiv Nadar Institution of Eminence, Delhi-NCR, India; 2 Special Centre for Molecular Medicine, Jawaharlal Nehru University, New Delhi, India; 3 Section of Microbiology, Central Ayurveda Research Institute Jhansi, Jhansi, Uttar Pradesh, India; 4 TCI Foundation, Gurugram, Haryana, India; 5 Tropical Animal Genetics Pvt Ltd., SPIC Bioprocess Laboratory, Anna University, Chennai, Tamil Nadu, India; 6 Amity Institute of Neuropsychology & Neurosciences, Amity University, Noida, Uttar Pradesh, India; IRCCS Sacro Cuore Don Calabria Hospital, ITALY

## Abstract

Potent inflammatory responses from host-parasite interactions in lymphatic filariasis are driven by macrophage polarization, which critically determines parasite survival or clearance. Evidence suggests that filarial parasite promote alternative macrophage polarization, facilitating immune evasion and persistent infection. However, the precise molecular mechanisms underlying filaria-induced alternative macrophage activation remain to be fully elucidated. Recently, serine protease inhibitors (serpins) have been implicated in alternative immune activation. Building on this insight, we explored and identified putative filarial serpins to be highly expressed in the infective L3 larval stage using *in-silico* analysis approach. Among all, Wb123, a serpin of *Wuchereria bancrofti*, the most predominantly found filarial worm, was cloned and purified to establish its role in alternative activation. We observed elevated markers of alternative activation; namely CD163, arginase-1, IL-6 and pSTAT3 expression, following rWb123 treatment. Furthermore, our results also indicated that rWb123 interacts with urokinase plasminogen activator receptor (uPAR) to activate the alternative activation pathway. Interestingly, rWb123 treatment attenuated the classical macrophage activation induced by lipopolysaccharide (LPS) and interferon-gamma (IFN-γ) as evident from muted CD86, nitric oxide (NO) and reactive oxygen species (ROS) expression. Notably, use of monoclonal antibody (MAbG8) to rWb123 or blocking uPAR impedes the rWb123-induced alternative activation and rescues the proinflammatory response to LPS-IFN-γ. These data confirmed that, uPAR dependent alternative activation by Wb123 enables filarial parasites to evade a strong pro-inflammatory immune response. Thus, targeting filarial serpins or uPAR could be potential therapeutics to re-establish immune response and eliminate filarial parasite from host.

## Author summary

Filarial nematodes are thread-like parasitic worms that causes major tropical diseases such as lymphatic filariasis and onchocerciasis. Macrophages of filariae infected individuals exhibit an alternatively activated phenotype. While previous studies have demonstrated that infective L3 larva infection and *Brugia malayi* antigen extracts can induce the alternative macrophage activation, the molecular mechanisms governing this process remains incompletely understood. In this study, we investigated the role of filarial serpin in modulating macrophage activation. We showed that recombinant Wb123 incubation induces the alternative macrophage activation through urokinase plasminogen activator receptor (uPAR) pathway. We also showed that pre-polarization of macrophages toward classical activation did not prevent subsequent Wb123-driven alternative activation. Additionally, neutralization of Wb123 with a monoclonal antibody reversed the alternative activation phenotype. Thus, these findings offer new insights into the immunomodulatory strategies employed by filarial nematodes and highlights the role of filarial serpins. They also open promising avenues for the development of therapeutic interventions targeting uPAR and serpins.

## 1. Introduction

Lymphatic filariasis is primarily caused by worms such as *Wuchereria bancrofti*, *Brugia malayi* and *Brugia timori*, while filarial worms like *Onchocerca volvulus* and *Loa loa* causes subcutaneous filariasis [1]. Lymphatic filariasis is a neglected tropical disease endemic to 39 countries, affecting approximately 36 million people with chronic disease manifestations and putting an additional 657 million at risk of infection [2]. These worms can live for about 6–8 years, producing millions of microfilariae that migrate to various organs, including the brain [3,4]. Microfilaria elicit a modified type 2 immune response characterized by the production of cytokines like IL-4, IL-10 and IL-13 along with the expansion of regulatory T cells [5,6]. Further studies on infective stage (L3 larvae) have shown reduced CD4 + T cell activation due to decreased ability of Langerhans’ cells (resident macrophages), indicating altered antigen-presenting function [7]. Macrophages are an important class of antigen-presenting cells that play crucial role in providing protection against pathogens by producing nitric oxide and other mediators [8]. They alter their phenotype and function in response to microenvironment stimuli, acquiring either a classically or alternatively activated phenotype [9]. During filarial infection, macrophages modulate the host immune responses, acting in a dual capacity. Studies involving *in-vitro* stimulation with filarial lysate, as well as analyses of macrophages from asymptomatic filarial patients demonstrates a predominance of alternatively activated macrophages associated with anti-inflammatory and regulatory functions [5,10]. In contrast, investigations on proteins isolated from the sheath of microfilariae and adult worms have also highlighted their role in driving pro-inflammatory response via classical macrophage activation [11,12]. Additional studies have demonstrated that monocytes of filarial patients are persistently exposed to parasite-derived excretory-secretory products (ESPs), which have been shown to significantly modulate lymphatic function and alter monocyte activity [13,14]. A comparative analysis of ESPs obtained from microfilariae, male and female worms revealed that homologues of serine protease inhibitors (serpins) were highly abundant in microfilaria ESP [15,16]. Serpins are a superfamily of protease inhibitors possessing a highly conserved secondary structure averaging 350–400 amino acids with a distinct reactive center loop (RCL). RCL of serpins contains recognition sites (P1-P1’) that determine the specificity for serine proteases [17,18]. Recently, a study identified a *Trichinella spiralis* serpin and highlighted its role in the alternative activation [19]. This discovery raises questions about the functional roles and molecular mechanisms of filarial serpins. Consequently, we investigated the role of filarial serpins as modulators of the host immune response.

## 2. Methods

### 2.1. Ethics statement

We followed all relevant ethical guidelines and patient data were anonymized to safeguard privacy. Ethical approval for the collection of patient blood samples was obtained from the review board of TCI (Transport corporation of India) foundation (NBS_Godda- 2023-24-32859).

### 2.2. Culturing of human brain macrophage

Human brain macrophage cells HMC3 (Human microglia clone 3, CRL-3304, ATCC) were cultured in Eagle’s Minimum Essential Medium (EMEM), supplemented with 10% fetal bovine serum (FBS), 1 mM Sodium Pyruvate, 1x non-essential amino acids (NEAA) and 0.1% penicillin-streptomycin. RAW 264.7 cells were cultured in 10% fetal bovine serum (FBS) Dulbecco’s Modified Eagle’s Medium (DMEM) medium. Cell cultures were maintained in a 25 cm^2^ Corning flask under humid conditions at 37 °C with 5% CO_2_. Sub-confluent HMC3 cells were passaged every second day using 0.5% Trypsin-EDTA. To induce classical activation of microglia, LPS (1ug/ml) was used along with IFN-γ (100 ng/ml). All experiments were performed within 4–15 passages.

### 2.3. Isolation of PBMCs from filarial patients

After initially screening fifteen individuals from the Govindpur region of Dhanbad district in Jharkhand, a field-based immunochromatographic card test for circulating filarial antigen was performed using whole blood collected from five individuals between 9 p.m. and midnight. Microscopic examination of calibrated thick smears resulted in the identification of two microfilaria-positive (MF+) and three microfilaria-negative (MF-) samples. Peripheral blood mononuclear cells (PBMC) from the blood of MF+ filarial patients and endemic normal donors were isolated using Ficoll density centrifugation (Hi-Sep LSM 1077). Trizol reagent was added to isolate RNA and cDNA was made to be subsequently used in qPCR-based analysis. All study participants provided informed written consent and were examined as a part of protocols approved by review board of TCI (Transport Corporation of India) foundation (NBS_Godda- 2023-24-32859).

### 2.4. Cloning and purification of rWb123 and antibody production

The Coding sequence (CDS) of the Wb123 gene was codon-optimized, synthesized de novo, and subcloned into NcoI and XhoI digested pET-28a (+) vector and transformed in E. coli Rosetta (DE3) cells. The expression of recombinant protein was induced using 1mM IPTG at 18 °C for 16 hours. The cell biomass was pelleted at 8000 g for 10 minutes, followed by lysis and sonication in resuspension buffer (30 mM Tris-Cl, 0.5 mM EDTA pH 7.5) until the viscous fluid became clear. Inclusion bodies were pelleted and washed repeatedly with buffer (50 mM Tris-Cl and 100 mM NaCl, 0.5% triton-100 and 0.1% sodium azide) followed by a final wash with buffer containing 50 mM Tris (pH 8.0) and 500 mM NaCl. The pellet was dissolved in a buffer containing 6 M GuHCl, 10 mM Tris (pH 8.0), 300 mM NaCl at room temperature for 16 hours. Following incubation, cells were centrifuged (Thermo-scientific, sorvall, LYNX-4000) at 13,000 rpm for 30 minutes at 4 °C. Ni-NTA beads were incubated with the supernatant on a rotor shaker overnight at 4 °C. The resins were packed into a column and washed with ten volumes of 8 M urea, 20 mM Tris-Cl and 500 mM NaCl (pH 8.0) containing 20 mM imidazole. Protein elution fractions were collected at imidazole concentrations of 50 mM, 150 mM, 250 mM and dialyzed in 20 mM Tris-HCl with NaCl at 150 mM (pH 7.4), 1 mM PMSF. The dialyzed protein concentration was determined by BCA assay and then run on a 15% SDS-PAGE.

For anti-Wb123 polyclonal antibody production, male BALB/c mice were injected subcutaneously with an antigen emulsion containing 50 µg of recombinant proteins and Complete Freund’s Adjuvant. Mice received three booster doses of a 25 µg antigen dosage in Incomplete Freund’s Adjuvant at intervals of 14 days. Pre-immune sera (0.5 ml) were collected on day 0 and the final bleed was collected on day 62, while the monoclonal antibody against Wb123 was outsourced from tropical animal genetics.

### 2.5. Immunofluorescence assay

To analyze the intracellular expression of proteins after treatment with rWb123 and LPS/IFN-γ in different combinations (Table 1), cells were seeded on the coverslips at a density of 1.5 x 104 in 24 well plate (NEST, tissue culture). After treatment, cells were fixed by 4% paraformaldehyde followed by permeabilization using 0.10% Triton X-100 and blocking with 2% BSA for 1 h prepared in 1x PBS. Rabbit raised primary antibody dilutions were incubated overnight at 4 °C. Alexa Fluor-488 conjugated anti-rabbit were used as secondary antibody. DAPI antifade (Vectashield, #H-1200-10) mounting media was used to mount the coverslips on the slide. Similarly, microfilaria positive (MF+) filarial blood samples were diluted with 1xPBS. Cells were pelleted and fixed. Blocking was performed for 2 hours at RT. Both primary monoclonal and polyclonal antibodies against Wb123 were used followed by secondary Alexa Fluor-488 (Biorad, #5196-2404) conjugated antibody for detection, staining and visualization. Blood smears were mounted and images were captured using a Nikon Ti2 confocal microscope.

**Table 1:** Experimental strategies for marker expression analysis.

S. No.	Treatment (5 Hours)
1.	Control: Media
2.	Condition 1: rWb123
3.	Condition 2: LPS and IFN-γ (LPS-I), 1 ug/ml-100 ng/ml
4.	Condition 3: LPS-I + rWb123, 250 ng

### 2.6. In silico screening- Molecular docking and simulation analysis

The proteome of filarial worms was compiled using FASTA protein files of *Wuchereria bancrofti* (BioProject-PRJEB536), *Brugia malayi* (BioProject-PRJNA10729), *Brugia timori* (BioProject-PRJEB4663) and *Onchocerca volvulus* (BioProject-PRJEB513), downloaded from WormBase Parasite database. The raw HMM sequence for serine protease inhibitors was obtained from the Pfam webserver (PF00079) and a standalone BLAST search was conducted against the compiled database. Multiple sequence alignment (MSA) was performed using Clustal omega [20]. The predicted structure of Wb123 (Uniprot: F6KME1) was retrieved from AlphaFold Protein Structure Database and validated through a Ramachandran plot (Saves-v6.1) and ProSA-web server. The crystal structure of human uPA (PDB ID: 4DVA) at 1.94 Å resolution was retrieved from the Protein DataBank (PDB). Molecular docking of Wb123 and uPA was performed by using HDOCK server. Protein-protein complex interactions were evaluated with the PDBsum and LigPlus software. The uPA-Wb123 complex stability was analyzed using GROMACS 5.1.5 through the LiGRO tool. The system was prepared in a TIP3P water model with 0.15 M NaCl in a cubic box (4 nm periodic image distance) using the Amber99sb force field. System equilibration involved NVT and NPT ensemble runs (1000ps each) using Berendsen thermostat and Parrinello-Rahman barostat. A 150ns production MD was performed at 310.15 K and 1.01 bars. The simulation employed PME for electrostatics, LINCS for hydrogen bonds, and a 2 fs time step with 1ps frame storage. Analysis utilized PyMOL and VMD for visualization, standard GROMACS tools for stability metrics, and gmx_MMPBSA tool for binding energy calculations from the final 150 frames.

### 2.7. Antibody-dependent blocking of urokinase plasminogen activator receptor

To evaluate the effect of uPAR blocking on rWb123 function, we pre-incubated cells in one well with uPAR antibody (DF12495) for 2 hours. rWb123 was incubated for 5 hours. After incubation, cells were lysed using RIPA buffer and run on 12% SDS-PAGE to evaluate CD163 expression using immunofluorescence and western blot assay.

### 2.8. Western blotting

To investigate expression of pro-inflammatory and anti-inflammatory markers under conditions described in Table 1. Cells were lysed using RIPA buffer containing 1x Protease inhibitor cocktail (Roche). Protein estimation was performed using the BCA assay (Sigma, #QPBCA-1KT). Equal amount of protein from each sample were separated on 12% SDS-PAGE (Biorad) and was transferred to the PVDF membrane using Trans-blotter (Biorad). The blots were evaluated for protein expression using primary antibodies listed as Table B in S1 Text followed by 1 h incubation with anti-rabbit HRP conjugated secondary antibody (1:5000) incubation. GAPDH was used as loading control. Blots were developed in iBright (Invitrogen) using ECL substrate (Biorad #1705061).

### 2.9. Enzyme-Linked immune sorbent assay (ELISA) to evaluate MAbG8 interaction with Wb123

To assess the interaction of rWb123 with MAbG8, a 96-well ELISA plate was coated with purified rWb123 (bait; 100 ng of each) in PBS at RT for 5 h, blocked with 5% BSA in PBS overnight at 4°C. Further, increasing concentration of MAbG8 was added and incubated for 2 h at RT. After washing with PBS, secondary anti-mice HRP conjugated antibody was incubated at RT for 2 h. Detection reagent (TMB; HIMEDIA) was added, after washing with PBS, the HRP reaction was stopped by adding 3 M HCl. Absorbance was measured at 450 nm using a microplate reader and graph was plotted using GraphPad PRISM software.

### 2.10. Antibody neutralization experiment

To evaluate the effect of anti-Wb123 monoclonal antibody (MabG8) on rWb123 induced alternative activation, we incubated anti-Wb123 antibody (1:5) with 1 ug rWb123 and alone rWb123 was used as control. Next day, rWb123-MAbG8 complex and rWb123 were added to the cells in EMEM media for 5 hours. Then, cells were either fixed using 4% paraformaldehyde for immunofluorescence study or lysed for western blot analysis as described earlier. Similarly, to evaluate the effect on ROS production, cells were treated with LPS/IFN-γ for 3 hours before addition of Wb123-MAbG8 complex and rWb123 in EMEM media. 5 µM carboxy-H2DCFDA (Invitrogen catalog #136007) was added to the cells in Opti-MEM media for 20 minutes and visualized using confocal microscope. We also evaluated the uPA expression using anti-uPA antibody (1:200).

### 2.11. Study ROS and CD163 by flow cytometer

For ROS expression analysis cells were given treatment as described in Table 2. After treatment cells were trypsinized and centrifuged (Gyrogen-1580R) at 850 rpm for 5 mins followed by wash using 1x PBS. Afterwards cells were suspended in 200 ul of 1x PBS and 100 nM carboxy-H2DCFDA (Invitrogen, #136007) and added to the 1.5 ml microcentrifuge tubes. We used 20 µM H_2_O_2_ (10 min) as a positive control for the induction of ROS and no-dye control to nullify the background noise in our experiment. We performed flow cytometry in Beckman Cytoflex flow cytometer (10000 events captured) and CytExpert was used for analysis. Similarly, following a one-hour incubation with 30 µg/ml of the NF-kB peptide inhibitor (SN50i) and TLR4 peptide inhibitor (TIRAPi), which target NF-kB and TIR adapter protein respectively, cells were treated with rWb123 for 5 hours, followed by washing with 1x PBS. Cells were trypsinized and processed for FACS analysis using the immunofluorescence protocol described above.

**Table 2:** Flowcytometry strategies for ROS expression.

S. No.	Treatment (5 Hours)
1.	Condition 1: Media+ 5 µM carboxy-H_2_DCFDA
2.	Condition 2: LPS-I (3 hrs), 5 µM carboxy-H_2_DCFDA
3.	Condition 3: LPS-I (3 hrs) + rWb123 (5 hrs), 5 µM carboxy-H_2_DCFDA
4.	Condition 4: LPS-I (3 hrs) +rWb123 + MabG8 (5 hrs), 5 µM carboxy-H_2_DCFDA

### 2.12. Q-PCR-based gene expression study

Microglia cells after 5 hours treatment in various experimental conditions were trypsinized and pellet down to isolate RNA. TRIZOL reagent (Thermo fisher, #15596206) was used and the RNA concentration was measured using the Nanodrop 2000 instrument (Thermo-Scientific). The RNA was used to make complementary DNA (cDNA) using cDNA reverse transcription kit (Applied Biosystems, #4368814). Powerup SYBR green master mix (Applied Biosystems # A25742) and gene-specific primers were used to perform quantitative PCR in AB-Step OnePlus instrument. Fold change (2-ΔΔCt) calculations were performed in Microsoft excel, normalized with the housekeeping gene 18S. The bar plot was plotted using GraphPad Prism 9 software. The qPCR primers are mentioned (Table C in S1 Text).

### 2.13. uPA inhibition assay

Inhibition of uPA activity was tested by performing direct fluorescent assay. Here, serial dilutions of rWb123 were added to the wells of 96-well plate (black) on ice containing recombinant uPA from human kidney cells (Cat# SRP6273, Sigma-Aldrich) and urokinase fluorogenic substrate III (Z-Gly-Gly-Arg-AMC, Cat# 672159, Sigma-Aldrich) to make a final volume of 200 μL/well, final enzyme concentration 2 nM, and final substrate concentration 250 μM in assay buffer (20 mM HEPES pH 7.4, 100 mM NaCl, 0.5 mM EDTA, 0.01% v/v Tween-20). Reaction was monitored immediately following addition of the enzyme using a microplate fluorescence reader (BioTek, Synergy H1) at 37 °C using excitation wavelength (365–380 nm) and emission wavelength (430–460 nm). Significant inhibition of the uPA activity by the different rWb123 concentrations was calculated using uPA without rWb123 as 100 percent activity. Recombinant PAI1(Cat# A8111, Sigma-Aldrich) was used in 1:1 ratio to uPA as positive control. Graphed using GraphPad PRISM v8.0 software.

## 3. Results

### 3.1. *In-silico* screening identifies filarial serpins highly expressed in infective L3 larval stage

**Fig 1:**
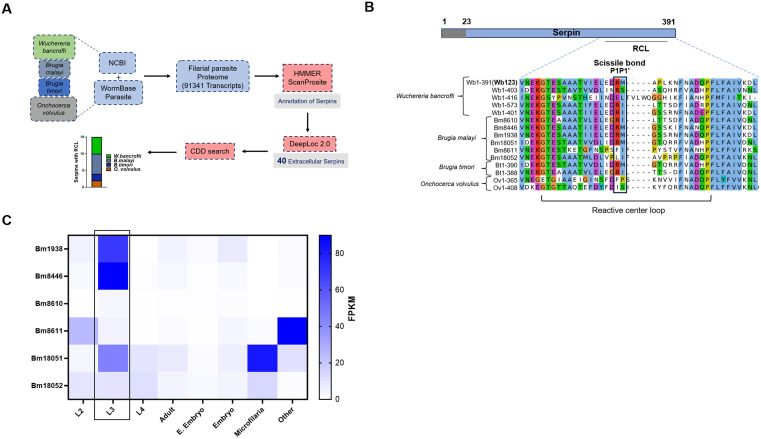
*In-silico* study identifies filarial serpins and target serine protease. **A)** Schematic representation of the pipeline devised to screen filarial serpins; **B)** Multiple sequence alignment of RCL for Wb123 and other identified filarial serpins. P1-P1’ sites are shown in the box; the First 2 letters in each serpin represent abbreviations of the scientific names of given species: Wb, *W. bancrofti*; Bm, *B. malayi*; Bt, *B. timori*; Ov; *O. volvulus*; **C)** Heatmap represents the expression of identified *Brugia malayi* serpins in different life stages from WormBase expression database.

A previous study reported that serpins are part of excretory and secretory products of filarial worms [16]. We performed virtual screening using a filarial proteomic database containing 91341 transcripts. To identify extracellular filarial serpins, we began by annotating a pool of proteins for the serpin signature motif (PS00284, PF00079). A total of 66 “high confidence” serpin proteins were retrieved which were further annotated for subcellular localization resulting in identification of 40 extracellular serpins. We conducted conserved domain analysis on 40 predicted extracellular serpins to identify serpins with RCL, a structural element critical for binding to serine proteases. This screening revealed 15 filarial serpins exhibiting conserved RCL (Fig 1A). Interestingly, comparative analysis of RNA-seq data derived from multiple developmental stages of *Brugia malayi* obtained from WormBase, revealed elevated expression levels of identified filarial serpins during the infective L3 larval and microfilariae stages. This expression pattern aligns with previous reports demonstrating significant upregulation of these serpins in microfilariae relative to other developmental stages (Fig 1C) [16,21]. Such stage specific enhanced serpin expression suggested their potential role in immune evasion during transmission and establishment phases. Additionally, we performed MSA of RCL to annotate P1:P1’ residues (scissile bonds) known for their specificity for protease targets. This analysis revealed that 10 out of the 15 serpins have Arginine as a P1 residue, and 5 of these serpins possess both Arginine and Methionine as a P1:P1” residues (Fig 1B). Previous studies have shown that Arginine and Methionine as a P1:P1” residues are common for serpins targeting serine protease plasminogen activator [17,18]. These results suggest that filarial serpins could target plasminogen activator, which is crucial for activating the complement system and mounting an inflammatory response [22–24].

### 3.2. Serine protease plasminogen activator identified as interacting partner of filarial serpin Wb123

**Fig 2:**
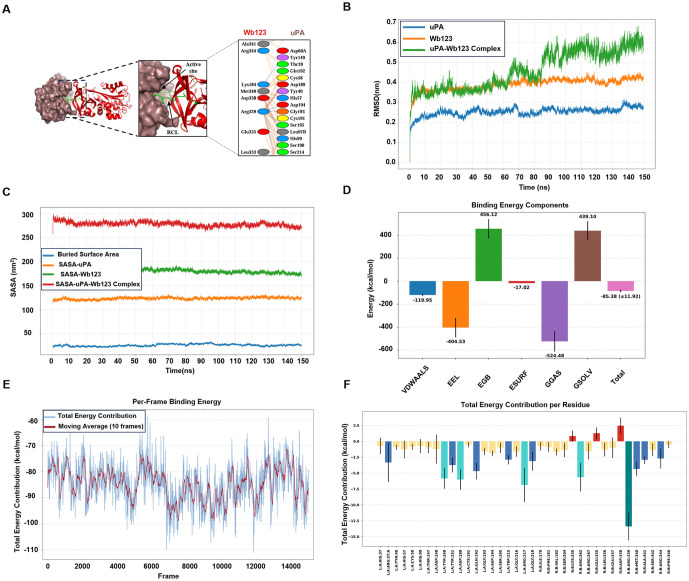
Docking and molecular dynamics simulations show stable Wb123-uPA complex formation. **A)** Biophysical interactions between filarial serpin Wb123 (shown in red) and uPA (shown in brown). Zoom-in showing the binding interface between Wb123 RCL residues (Arg359, Met360) and uPA catalytic site (His57, Asp189, Ser195), **B)** RMSD plots for uPA, Wb123, and the uPA-Wb123 complex. **C)** Solvent Accessible Surface Area (SASA) of uPA, Wb123, and the uPA-Wb123 complex, alongside the buried surface area at the interface. **D)** Contributions of individual energy components to the total MMGBSA binding energy, VDWAALS-Van der Waal energy; EEL-Electrostatic energy; EGB-Polar solvation energy; ESURF-Non polar solvation energy; GGAS-Gas phase free energy; GSOLV-Solvation free energy. **E)** Per-frame MMGBSA binding energy calculations with a superimposed moving average, showing binding affinity fluctuations over time. **F)** Per-residue energy contributions to the binding energy, highlighting key residues involved in the interaction.

Considering that *Wuchereria bancrofti* accounts for more than 90% of filarial cases, we selected the serpin Wb1–391 for further characterization and named it Wb123. The full-length sequence of Wb123 has 391 amino acids, with a theoretical molecular weight of 44 kDa and an isoelectric point (PI) of 8.43. Further sequence analysis revealed that wb123 contains a serpin domain (cd00172) with a conserved RCL at positions 345–371 aa and an exposed putative nuclear localization signal (PKRRFG) at positions 254–259 aa. The modelled structure of Wb123 was validated using the SAVESv6.1 and ProSA web server, yielding an ERRAT score of 93.39 (S1A and S1C Fig). Ramachandran plot indicated that 92.2% (319aa) of residues were in the most favored regions, 6.9% (24aa) in the additionally allowed regions, 0.6% (2aa) in generously allowed regions and 0.3% (1aa) in the disallowed region, with z score of -8.4 (S1B Fig). Furthermore, the modelled structure of Wb123 was superimposed on resolved structures of PAI-1, Alpha-1-Antitrypsin, Glia-derived nexin and kallistatin showing RMSD values of 1.530, 1.687, 1.611 and 1.845 respectively, confirming structural similarities with human serpins (Table A in S1 Text). To investigate serine protease target of filarial serpin Wb123 we performed molecular docking with human serine proteases such as cathepsin G (CTG), plasmin (PL), elastase (EL) and urokinase plasminogen activator (uPA). Docking scores suggested that Wb123 exhibited the highest binding energy of -387.17 for uPA compared to other serine proteases, CTG (-365.01), PL (-295.62) and EL (-308.57). Notably, the RCL was only involved in Wb123-uPA binding (S2A Fig). Interaction analysis of Wb123 and uPA complex revealed that amino acid residues arginine-339 and methionine-340 (p1-p1’) formed a stable complex with the catalytic triad (His57-Ser195-Asp189) of uPA, stabilized by salt bridges and hydrogen bonds (Figs 2A and S2C), similar to PAI-1-uPA complex (3PB1) a known human serpin targeting uPA (S2B Fig) [25]. To assess the structural stability and interaction dynamics of the uPA-Wb123 complex, we performed molecular dynamics simulation (MDS) trajectory analyses focusing on root mean square deviation (RMSD), solvent-accessible surface area (SASA), and binding free energy. RMSD profiles were compared for uPA, Wb123, and the uPA-Wb123 complex. The uPA protein exhibited notable structural stability, maintaining an RMSD of approximately 0.25 nm after the initial 10 ns. In contrast, Wb123 displayed higher conformational flexibility, with RMSD values approaching 0.4 nm. The complex exhibited a higher RMSD range of 0.50-0.60 nm after 90 ns, indicative of dynamic rearrangements while retaining a stable binding conformation (Fig 2B). These results highlight that the uPA-Wb123 interaction remains stable despite the intrinsic flexibility of Wb123. SASA analysis for both uPA and Wb123 in complex form revealed minimal fluctuations, alongside a reduced buried surface area, consistent with effective molecular interactions upon complex formation (Fig 2C). Further, binding free energy calculations using the Molecular Mechanics Generalized Born Surface Area (MM-GBSA) approach yielded a value of -85.38 ± 11.92 kcal/mol representing a highly stable and energetically favorable uPA-Wb123 interaction (Fig 2D). Per-frame energy analysis revealed that the average binding energy remained relatively constant throughout the simulation, with only minimal fluctuations, further supporting the stability inferred from RMSD data (Fig 2E). Additionally, per-residue interaction energy analysis identified key contributors to the uPA-Wb123 interface specifically, residues His57, Tyr149, Tyr151, Asp189, Gly193, Ser195, Arg217, and Gly219 from uPA were found to interact actively with Arg262, Arg339, Met340, Ala341, Ser342, and Arg344 of Wb123 (Fig 2F). Notably, His57, Ser195, and Asp189 constitute the catalytic triad of uPA, while Arg339 and Met340 represent the P1 and P1′ residues within the reactive center loop of Wb123, functioning as bait in complex formation. Lastly, visualization of three-dimensional structures further highlighted the spatial arrangement and nature of these intermolecular contacts, corroborating the observed thermodynamic stability of the Wb123-uPA complex throughout the simulation (S2D Fig). Collectively, these integrated analyses provide a comprehensive characterization of the structural dynamics and energetic landscape governing the Wb123-uPA interaction.

### 3.3. Recombinant Wb123 induces alternative activation of macrophages

**Fig 3:**
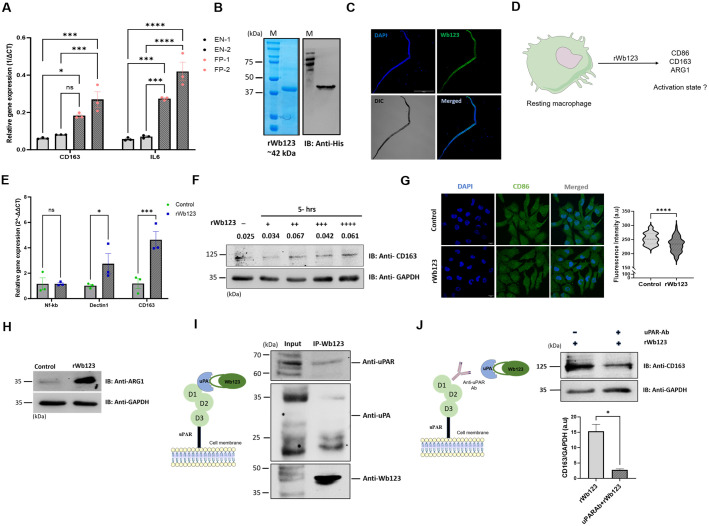
rWb123 induces alternative activation through uPAR. **A)** CD163 and IL-6 expression in MF+ filarial patients. The graph represents fold change in gene expression of filarial patient compared with endemic normal. Data represent the Mean ± SEM (n = 3). *represents significance values compared to control, *p<0.0214, **p=0.0012, ***p=0.0006 and 0.0001, ****p<0.0001. Statistical significance was calculated using two-way ANOVA. **B)** SDS-PAGE stained with Coomassie brilliant blue dye and western blot analysis of purified rWb123 with His tag antibody. **C)** Images showing Wb123 expression in microfilaria probed with monoclonal antibody. **D)** Strategy for evaluating rWb123 role in macrophage activation. **E)** Study of activation marker expression after incubation with rWb123. The graph represents fold change in gene expression. Data represent the Mean ± SEM (n = 3). *represents significance values compared to control, *p<0.0194, ***p=0.0002 and 0.0005. Statistical significance was calculated using two-way ANOVA. **F)** Blot showing CD163 expression in rWb123-treated cells, compared to control cells, across varying rWb123 concentrations. The concentrations are denoted as +(125 ng), ++(250 ng), +++(500 ng), and ++++(1 µg). CD163 expression levels are quantified as the CD163/Gapdh ratio. **G)** Images showing CD86 expression in rWb123 treated cells and untreated cells; Intensity was determined from cells (Violin plot, n = 70, *represents significance values compared to control, *P<0.0107, ****p<0.0001). Statistical significance was calculated using unpaired t test. **H)** Blot showing arginase-1 expression in rWb123 treated cells and untreated cells. **I)** Scheme representing interacting partners of Wb123. Immunoprecipitation analysis showing uPA and uPAR interaction with rWb123. **J)** Strategy for antibody blocking experiment for CD163. Blot showing CD163 expression in uPAR blocked cells and control cells. The bar graph represents quantification of CD163 expression in uPAR pre-treated and untreated cells. Normalized to GAPDH (Mean ± SEM, n = 3, AU depicts arbitrary unit, *represents significance values compared to control, *p<0.00307).

Patients with filarial infection exhibit a compromised immune response characterized by a diminished inflammatory response [10]. To understand the activation status of macrophages in filarial patients, we performed qPCR analysis and our results from PBMCs of microfilaria-positive (MF+) patient samples (FP-1 and FP-2) demonstrated increased CD163 and IL-6 expression compared with endemic normal control (EN-1 and EN-2), suggesting alternative activation (Fig 3A). Furthermore, we analyzed the publicly available gene expression database which revealed that filarial patients exhibited significantly higher levels of alternative activation markers, which include CD163, ARG-1, IL-4 and TGF-β while showing reduced expression of classical activation markers such as CD14 and CD86, solidifying the alternative macrophage activation (S3A Fig).

Serpins not only acts as inhibitors of serine proteases but have also evolved alternative, non-inhibitory roles [26]. To investigate its function recombinant Wb123 (rWb123) was expressed in *Escherichia coli* as a 6x his tagged protein and purified by Nickel NTA affinity chromatography. The molecular weight of purified rWb123 was observed at 42kDa on SDS PAGE, confirmed through western blot analysis (Fig 3B). Next, we confirmed the identity of the purified protein through mass spectrometry and the secondary structure analysis was performed using circular dichroism (CD) spectroscopy confirming that the protein was properly folded (S3B and S3C Fig). Furthermore, we detected the expression of Wb123 in the outer membrane of microfilaria using monoclonal anti-Wb123 antibody (Fig 3C).

To investigate the effect of recombinant filarial serpin rWb123 on macrophage activation, we incubated macrophages with rWb123 for 5 hours and subsequently analyzed the expression of alternative activation markers using qPCR (Fig 3D). Specifically, we assessed CD163, a hemoglobin-haptoglobin scavenger receptor and Dectin-1, a principal receptor for β-glucans on macrophages. Both CD163 and Dectin-1 are upregulated by cytokines that drive alternative macrophage activation and are typically downregulated by LPS [27,28]. Our qPCR data revealed that rWb123 treatment led to increased expression of CD163 and Dectin-1 compared to control cells, while NF-kB expression remain unchanged (Fig 3E). Western blot analysis further confirmed a dose-dependent increase in CD163 protein levels following rWb123 exposure at concentrations ranging from 125 ng to 1 µg (Fig 3F). Furthermore, we assessed CD86 expression, a well characterized classical activation marker upregulated in response to LPS stimulation [29]. Our results revealed that rWb123 treated cells exhibited no detectable CD86 expression compared to control cells (Figs 3G and S4A). Given that the induction of arginase-1 is crucial for establishing alternative activation, we investigated its expression levels and observed a significant increase in arginase-1 compared to control cells confirming that rWb123 polarizes macrophage towards alternative activation (Figs 3H and S4B). Additionally, we used a recombinant non-serpin filarial protein (NSFP) as a negative control and observed that it did not induce CD163 expression (S4C Fig). Furthermore, we also evaluated CD206 expression in rWb123 treated mouse macrophages and observed increased expression compared to control cells (S4D Fig).

To identify the interacting partners of rWb123, we performed an immunoprecipitation assay using an anti-wb123 antibody followed by western blot analysis with Toll-like receptor 4 (TLR4), uPA and uPAR antibodies (Fig 3I). Our western blot results showed that rWb123 interacts with uPA and uPAR but not with TLR4. To assess whether the interaction between rWb123 and uPAR is critical for its function, we performed an antibody-blocking assay since uPAR is a GPI-anchored receptor. We observed that pretreatment of cells with a uPAR-specific antibody resulted in a significant reduction of CD163 expression, as demonstrated by both western blot and immunofluorescence analysis. (Figs 3J and S5A).

### 3.4. rWb123 mediated alternative activation is IL-6 and STAT3 dependent

**Fig 4:**
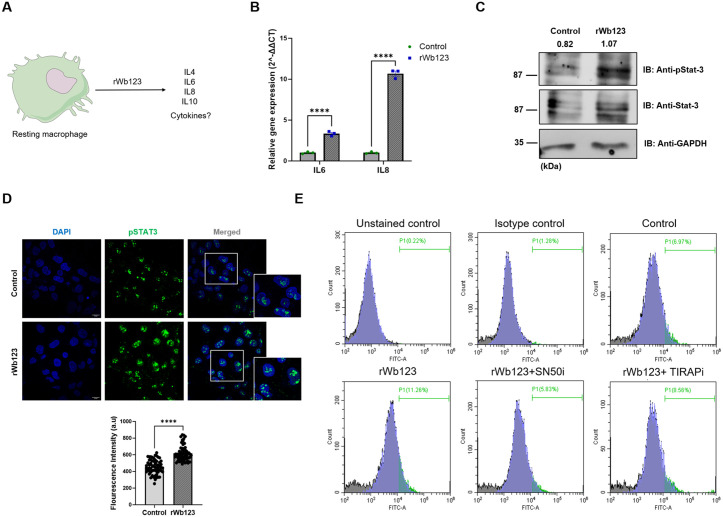
rWb123 induced alternative activation is IL-6-STAT3 mediated. **A)** Strategy for evaluating cytokines expression; **B)** The graph represents fold change in gene expression. Data represent the Mean ± SEM (n = 3). *represents significance values compared to control, ****p<0.0001. Statistical significance was calculated using two-way ANOVA; **C)** Blots showing phosphorylated STAT3 expression at protein level in rWb123 treated cells compared to control cells, pSTAT3 expression levels are quantified as the pSTAT3/GAPDH ratio.; **D)** Images showing STAT3 and phosphorylated STAT3 expression in rWb123 treated and untreated cells; Intensity was determined from cells (Mean ± SEM, n = 60, ****p<0.0001). Statistical significance was calculated using unpaired t-test; **E)** Histogram representing CD163 expressing in control, rWb123, TIRAPi and SN50i treated cells respectively.

Notably, cytokines such as IL-4, IL-10 and IL-13 are known to induce alternative activation of macrophages [30,31]. Thus, we also wanted to investigate the cytokines that play major role in Wb123-induced alternative activation (Fig 4A). Our qPCR results revealed a significant upregulation of IL-6 and IL-8 expression in rWb123-treated cells relative to untreated controls (Fig 4B), whereas IL-4 and IL-10 transcripts were undetectable under these experimental conditions. Since STAT3 is a downstream signaling target of IL-6, IL-8 and had been previously shown to play a major role in alternative activation [32–35]. We evaluated the effect of rWb123 on STAT3, our immunofluorescence and western blot results showed increased expression of phosphorylated STAT3 in rWb123-incubated cells compared to control cells (Fig 4C and 4D). Additionally, we confirmed that incubation with rWb123 at concentrations ranging from 125 ng to 1 µg induced a dose-dependent increase in STAT3 phosphorylation. (S5B Fig). Furthermore, to investigate the role of nuclear factor kappa-light-chain-enhancer of activated B cells (NF-kB) in rWb123-mediated CD163 expression, we employed SN50i, a cell-permeable NF-kB inhibitory peptide and performed flow cytometry. As anticipated, rWb123 treatment markedly increased the population of CD163-expressing cells compared to untreated controls, with mean fluorescence intensity (MFI) values of 32886 and 24454, respectively. Pre-treatment with SN50i, resulted in a 40–50% reduction in the CD163-positive cell populations, accompanied by a decreased MFI of 28359, highlighting the pivotal role of NF-kB in regulating CD163 expression (Fig 4E). Given the established role of NF-kB in LPS signaling, we examined the contribution of Toll/interleukin-1 receptor domain-containing adaptor protein by pretreating cells with TIRAPi, a cell-permeable inhibitor. Pretreatment with TIRAPi did not result in any significant change in CD163 expressing populations, a result further corroborated by western blot analysis (Figs 4E and S5C).

### 3.5. rWb123 impairs the LPS-IFN-γ induced classical activation

**Fig 5:**
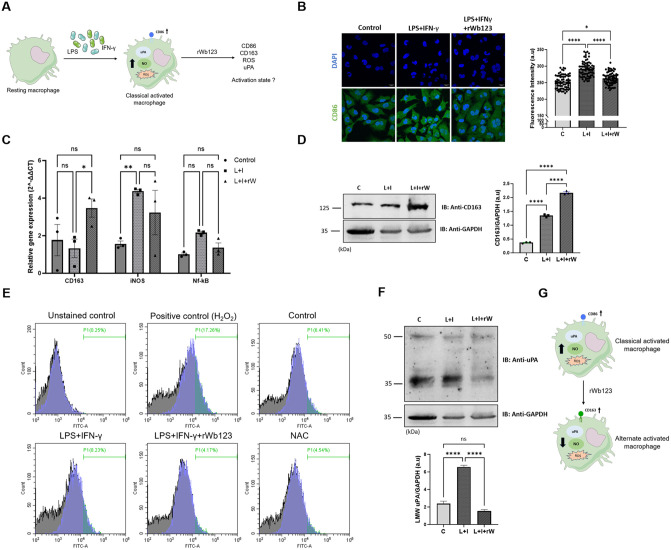
LPS-I induced classical activation is abrogated by rWb123. **A)** Strategy to evaluate rWb123 effect on LPS-I induced classical activation. **B)** Immunofluorescence images showing CD86 expression in control cells compared with LPS-I and LPS-I with rWb123, Intensity was determined from cells (Mean ± SEM, n = 70, *represents significance values compared to control, *P < 0.0132, ****p < 0.0001). Statistical significance was calculated using one-way ANOVA. **C)** Activation marker expression analysis in HMC3 cells treated with LPS-I alone and LPS-I along with rWb123. The graph represents fold change in gene expression. Data represent the Mean ± SEM (n = 3). *represents significance values compared to control, ****p < 0.0001. Statistical significance was calculated using two-way ANOVA. **D)** Blots showing CD163 expression in control cells compared to LPS-I alone and LPS-I with rWb123 treatments, The bar graph represents quantification of CD163 expression normalized to GAPDH (Mean ± SEM, n = 3, AU depicts arbitrary unit, *represents significance values compared to control, ****p < 0.0001). **E)** Histogram representing ROS in cells treated with LPS-I alone and LPS-I with rWb123, along with H2O2 and NAC as positive and negative controls, respectively. **F)** Blots showing uPA expression in LPS-I and LPS-I with rWb123 treated cells. the bar graph represents quantification of LMW uPA (~30kDa) expression normalized to GAPDH (Mean ± SEM, n = 3, AU depicts arbitrary unit, *represents significance values compared to control, ****p < 0.0001). **G)** Schematic representation of Wb123 effect on classical activated macrophages.

To evaluate the effect of rWb123 on classical activation, we pre-incubated cells with LPS and IFN-γ, collectively referred to as LPS-I, well-known inducers of classical activation [36]. This was followed by rWb123 treatment (Fig 5A). As expected, proinflammatory marker CD86 expression was higher in LPS-I treated cells compared to control cells. However, CD86 expression was decreased when LPS-I treated cells were incubated with rWb123 suggesting reduced classical activation (Fig 5B). Additionally, we evaluated the expression of alternative activation marker and our qPCR result indicated that CD163 expression was higher in LPS-I pre-treated cells after rWb123 treatment, validated through western blot analysis (Figs 5C, 5D and S6A). Given that rWb123 impaired the classical activation, we hypothesized that it might also influence the oxidative status of the cells. To evaluate this, we assessed reactive oxygen species (ROS) production by labelling cellular ROS with H_2_DCFDA, a fluorescent dye. Flow cytometry analysis demonstrated that LPS-I treatment induced a significant increase in the population of cells expressing reactive oxygen species (ROS+) with increased MFI value of 5110 compared to 4842 in control cells. In contrast, rWb123 treatment in LPS-I primed cells resulted in marked reduction of ROS+ cell populations (40–50%) lowering the MFI to 4063 and effectively restoring levels to those observed in untreated controls. As controls, we used 20 µM hydrogen peroxide (H_2_O_2_) which significantly elevated ROS+ populations with MFI value of 7719, while the potent antioxidant N Acetyl-cysteine (NAC) markedly decreased the ROS+ populations with MFI value of 4047 (Fig 5E). These findings indicate that rWb123 attenuates the LPS-I induced oxidative stress, yielding ROS levels comparable to baseline. We also evaluated the NO expression, using in-house probe and found that its expression was significantly decreased in cells incubated with rWb123, even after LPS-I activation (S6B Fig) [37]. Since, uPA expression has been correlated with the oxidative status of macrophages and is known to induce ROS production [38]. We evaluated the effect of rWb123 on uPA expression in LPS-I primed cells. Notably, our findings indicated that the expression of low molecular weight uPA, which was significantly increased following LPS-I treatment, was reduced in cells incubated with rWb123, even after activation by LPS-I which we further validated through immunofluorescence assay (Figs 5F and S6C). Together, these findings suggest that rWb123 not only suppresses classical macrophage activation but also mitigates associated oxidative stress and pro-oxidant uPA expression, thereby promoting a shift towards less inflammatory phenotype (Fig 5G).

### 3.6. Monoclonal antibody abrogates the effects of rWb123 on macrophage activation

**Fig 6:**
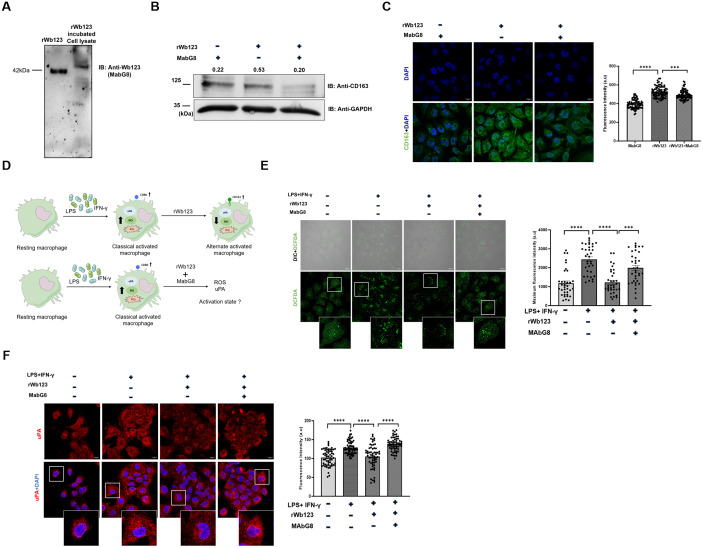
Monoclonal antibody MAbG8 impedes the Wb123-induced alternative activation. **A)** Western blot image showing rWb123 and rWb123 treated cell lysate probed with MAbG8. **B)** Blots showing CD163 protein expression in MabG8 treated cells, cells treated with rWb123 and rWb123 with MAbG8, CD163 expression levels are quantified as the CD163/Gapdh ratio. **C)** Images showing CD163 expression in MabG8 treated cells, cells treated with rWb123 and rWb123 with MAbG8. Intensity was determined from cells (Mean ± SEM, n = 80, ***p = 0003, ****p < 0.0001). Statistical significance was calculated using ordinary one-way ANOVA. **D)** Schematic representation of strategy employed for evaluating MabG8 effect on rWb123 function. **E)** Confocal microscopy images showing ROS expression in control cells compared with LPS-I, LPS-I with rWb123 and LPS-I with rWb123-MAbG8. Intensity was determined from cells (Mean ± SEM, n = 35, ***p = 0.0003, ****p < 0.0001). Statistical significance was calculated using ordinary one-way ANOVA. **F)** Images showing uPA expression in control cells compared with LPS-I, LPS-I with rWb123 and LPS-I with rWb123-MAbG8. Intensity was determined from cells (Mean ± SEM, n = 60, ^**ns**^ = 0.1120, ****p < 0.0001). Statistical significance was calculated using ordinary one-way ANOVA.

To investigate the potential of monoclonal antibody to block rWb123 function. We initially validated the specificity of monoclonal antibody ‘MabG8’ for rWb123 using both enzyme-linked immunosorbent assay (ELISA) and Western blot analysis (Figs 6A and S6D). Subsequently, we assessed its impact on rWb123 function by conducting an antibody neutralization assay. Our results indicated that overnight incubation of MabG8 with rWb123 significantly reduces rWb123-induced CD163 expression, with rWb123-treated cells serving as a control (Fig 6B and 6C). Additionally, to verify that MabG8 counteracts the alternative effects of rWb123, we examined its effect on cells activated by LPS-I (Fig 6D). Consistent with previous observations, our findings indicated that incubation of rWb123 alone resulted in decreased ROS expression. However, the co-incubation with MabG8 and rWb123, resulted in increased ROS expression (Fig 6E). Moreover, it also restored uPA expression, which had been suppressed by rWb123 alone (Fig 6F). Overall, our data illustrates that the monoclonal antibody MabG8 hinders the anti-inflammatory effects of rWb123.

## 4. Discussion

Filarial worms possess an extraordinary ability to modulate the host immune system, characterized by suppressed T cell activity and attenuated macrophage responses, suggesting interference at both cellular and molecular levels. Despite this, the precise mechanisms by which these parasites regulate macrophage responses to ensure their survival remain largely elusive. Several studies have sought to identify parasitic molecules involved in macrophage modulation from excretory-secretory products (ESPs) [15]. Recently, comparative analyses of ESPs from microfilariae, male, and female worms have revealed high expression of serine protease inhibitors (serpins) [16]. More recently, studies have also uncovered a role for serpins in promoting alternative macrophage activation. [19,32,39]. Serpins are a unique family of proteins that utilize a sophisticated “bait-and-trap” mechanism to inhibit serine proteases [18].

**Fig 7:**
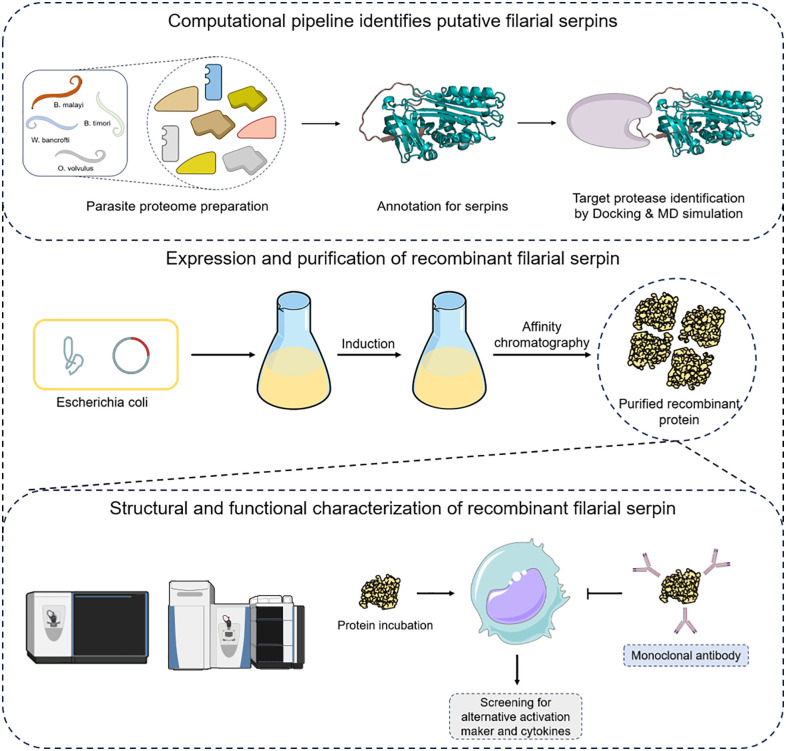
Schematic representation for the integrated experimental strategy.

Based on this background, we hypothesized that serpins in filarial worm might play important role in alternative macrophage activation. To prove this, we designed an integrated experimental strategy involving both *in-silico* and *in-vitro* applications as illustrated in the schematic representation (Fig 7). Thus, we performed *in-silico* screening and identified fifteen filarial serpins with conserved RCL which are highly expressed in L3 larval stage. Given that *Wuchereria bancrofti* accounts for the majority of cases and possesses only one serpin featuring an Arg-Met scissile bond, we selected serpin Wb123 for further study. After successfully purifying the rWb123, its sequence and structural integrity were validated by mass spectrometry and circular dichroism spectroscopy respectively. To evaluate Wb123’s role in macrophage polarization, we studied alternative macrophage activation markers such as Arginase-1 (a well-characterized indicator in parasitic infections), along with CD163 and Dectin-1, which have recently emerged as critical markers for characterizing this activation state [10,28,32,40–43]. Recent evidences indicates that Dectin-1 and CD163, although functionally distinct as pattern recognition receptors, may act in concert to regulate immune responses [44,45]. Our finding supports this concept as treatment with rWb123 induced the expression of CD163, Dectin-1, and Arginase-1, these changes in alternative activation markers were not observed with non-serpin filarial protein (NSFP) treatment. Furthermore, we also found increased CD163 and IL-6 expression in PBMC’s of microfilaria positive (MF+) filarial patients. This finding was further strengthened by our *in-silico* analysis of publicly available filarial patient datasets, which showed elevated expression of these alternative activation markers and decreased expression of classical activation markers in peripheral monocytes, with these changes reversing post-treatment [14].

Next, to identify the target protease of Wb123, we performed an *in-silico* analysis focusing on identifying the conserved domain and residues, critical for serine protease interactions. We found that serpins have a conserved RCL region containing scissile bonds (P1:P1’) that is known to act as a bait for the target proteases [17,18]. Considering this we performed domain analysis and found that the RCL of Wb123 features an Arg-Met scissile bond, exactly similar to serpin E1 (PAI-1) and serpin I1 (Neuroserpin), which are known inhibitors of the serine protease uPA [17]. Further, protein-protein docking and molecular simulation studies confirmed the interaction between Wb123 and uPA. Additionally, we performed interaction analysis of uPA-PAI-1 complex (3PB1) which revealed that similar to wb123, P1:P1’ residues of PAI1 were involved in the interaction with the catalytic triad of uPA, suggesting Wb123’s specificity toward uPA (S2B Fig).

Next, our results from fluorescence-substrate-based assay authenticated these interactions and suggested that rWb123 may inhibit uPA activity (S3D Fig). Additionally, immunoprecipitation assay substantiated this fact that rWb123 could interact with both uPA and uPAR, a crucial axis involved in orchestrating inflammatory signaling pathways. This data aligns with the existing evidence indicating that human serpin (PAI-1) and viral serpin (Ser-1) interacts with uPAR to activate downstream signaling responsible for alternative macrophage activation [32,39].

Building on this data, we conducted an investigation into the mechanistic role of Wb123/uPAR interaction in alternative macrophage activation. To elucidate uPAR’s involvement in Wb123-mediated alternative activation pathway, we blocked uPAR using a specific antibody, which resulted in significant reduction in CD163 expression, highlighting its critical function in rWb123-driven alternative macrophage activation. Importantly, although uPAR is a glycosylphosphatidylinositol (GPI)-anchored receptor incapable of direct signaling, it facilitates downstream signaling through interactions with vitronectin, integrins, and tyrosine/serine-threonine kinases [22,46–52]. Moreover, research has shown that uPAR knockout (uPAR-KO) mice exhibit severe inflammation, characterized by a pronounced shift in macrophage polarization towards the pro-inflammatory M1 phenotype [53].

Previous studies have demonstrated that the interaction of serpins with uPAR activates the p38MAPK/NF-kB/IL-6/STAT3 signaling loop, thereby promoting alternative macrophage activation [32,39]. This finding prompted us to investigate the cytokines involved in Wb123-driven alternative macrophage activation. Interestingly, instead of classical anti-inflammatory cytokines such as IL-4 and IL-10, we observed an increased expression of cytokines like IL-6 and IL-8. Although IL-6 is known for its diverse functions, emerging evidence highlights IL-6’s role in downregulating inflammation and facilitating alternative macrophage activation [33,54,55]. Recently, *in-vivo* and *in-vitro* studies have shown that IL-6 primes the macrophages toward IL-4 mediated alternative activation by inducing the expression of its receptor IL-4Rα through STAT3-mediated activation of the IL4ra promoter [56]. Taken together, these observations support the notion that IL-6 may play a role in early stages of alternative activation, ultimately paving the way for the induction of more prominent anti-inflammatory cytokines such as IL-4 and IL-10.

Next, we examined the activation status of STAT3, a key downstream signaling molecule of IL-4, IL-6, IL-10 and IL-13 [57–59]. Multiple studies have demonstrated that STAT3 promotes anti-inflammatory responses, whereas its absence leads to persistent classical macrophage activation and chronic inflammation [31,60,61]. Consistent with this, we observed increased levels of phosphorylated STAT3, reinforcing the role of IL-6-STAT3 signaling in driving alternative macrophage activation. In this context, it is worth highlighting the work of Kasmi and colleagues, who demonstrated that STAT3-mediated anti-inflammatory response is not exclusive to IL-10R signaling pathway but can also be triggered by any STAT3-activating cytokine receptors [61]. Furthermore, our results also demonstrated that Wb123-driven alternative macrophage activation, similar to PAI-1-induced activation, depends on NF-kB signaling, as its inhibition led to reduced CD163 expression.

Previously, studies have shown that LPS and IFN-γ (LPS-I) induces a robust classical macrophage activation characterized by the upregulation of reactive oxygen species (ROS), nitric oxide (NO), uPA and pro-inflammatory markers such as CD86 κ [62–67]. This led us to investigate the effect of rWb123 on LPS-I induced classical activation. In agreement to this, our results also suggested increased expression of ROS, NO, uPA and CD86 after LPS-I treatment however, rWb123 treatment attenuated the classical macrophage activation as evident from the muted expression of CD86, uPA, reactive oxygen species (ROS), and nitric oxide (NO). Conversely, the increased expression of CD163, suggests that pre-polarization of macrophages toward a classical phenotype by LPS-I does not preclude subsequent alternative activation in response to rWb123. Furthermore, blocking rWb123 with monoclonal antibody MAbG8 confirmed its role in alternative activation, as evidenced by decreased CD163 expression. Additionally, MAbG8-incubated rWb123 was assessed for its impact on LPS-induced classical activation. The antibody neutralized rWb123-mediated downregulation of uPA and ROS, with rescued expression observed upon MAbG8 treatment.

In conclusion, we identified and characterized a novel serine protease inhibitor (SERPIN) Wb123, which induces alternative activation of macrophages through a uPA/uPAR-dependent IL-6/STAT3 pathway substantiated by monoclonal antibody-based neutralization assay. Notably, Sharma et al. previously demonstrated increased IL-4 and TGF-β expression after day 3 of infection with *Brugia malayi* infective larval stage 3 (Bm-L3) and IL-10 expression after day 10 of infection suggesting alternative macrophage activation [68]. however, the specific parasite-derived molecules responsible for driving macrophage activation to evade host immune response remain unidentified. Our study provides novel insights into the molecular mechanisms driving macrophage polarization during the early stages of infection, opening new avenues for developing therapeutic strategies to prevent the establishment of filarial parasites. While *in-vitro* studies using *Wuchereria bancrofti* proteins yield important mechanistic insights, their direct physiological and therapeutic relevance remains challenging to establish, as the parasite exclusively infects human and no fully permissive experimental model that replicates the human host environment are available. Nonetheless, future investigations employing murine models of *Brugia malayi* infection could help elucidate the *in-vivo* consequences of filarial serpin neutralization, particularly when combined with uPAR receptor targeting, thereby enabling a more comprehensive evaluation of the therapeutic potential of these interventions.

## Supporting information

S1 Fig Post-modelling analyses of 3D structure of Wb123. **A)** Graph showing Errat score of modelled Wb123 structure. **B)** Ramachandran plot showing residue analysis of Wb123 structure done using PROCHECK server. **C)** Graph showing verify-3D score of Wb123 structure.
(TIF)

S2 Fig Interaction profile of Wb123 with serine proteases. **A)** Table representing the amino acid residues involved in the interaction of Wb123 with different serine proteases. **B)** Biophysical interactions between human serpin PAI-1(shown in blue) and uPA (shown in brown). Zoom-in showing the binding interface between PAI-1 RCL residues and uPA catalytic site. **C)** Graph showing number of hydrogen bonds during 150ns simulation of Wb123-uPA complex. **D)** Three-dimensional representation of the uPA-Wb123 interface, with residues colored blue indicating favorable binding contributions and red indicating unfavorable contributions.
(TIF)

S3 Fig Macrophage activation profile and biophysical assessment of recombinant Wb123. **A)** Heatmap represents expression of alternative and classical activation markers in pre-treatment, post-treatment and healthy person monocytes from gene expression browser. **B)** Image showing total ion chromatogram (TIC) and extracted ion chromatogram (XIC) of Wb123 from mass spectrometry analysis. **C)** Plot of circular dichroism spectra of Wb123 with secondary structure information. **D)** Bar graph showing uPA activity in varying rWb123 concentrations compared to control.
(TIF)

S4 Fig Assessing the effect of non-serpin filarial protein on CD163 expression. **A)** Blots showing CD86 expression in rWb123 treated cells, LPS-IFN-γ treated cells and control cells. **B)** Images showing Arg-1 expression in rWb123 treated cells and untreated cells. Intensity was determined from cells (Mean ± SEM, n = 70, *represents significance values compared to control, ****p < 0.0001), Statistical significance was calculated using unpaired t test. **C)** Blot showing CD163 expression in recombinant non-serpin filarial protein (rNSFP) treated cells, compared to control cells, across varying rNSFP concentrations. The concentrations are denoted as +(125 ng), ++(250 ng), +++(500 ng), and ++++(1 µg). CD163 expression levels are quantified as the CD163/Gapdh ratio. **D)** Blot showing CD206 expression in rWb123 treated mouse macrophages compared to control.
(TIF)

S5 Fig Wb123 treatment promotes the STAT3 phosphorylation and uPAR-dependent CD163 expression. **A)** Images showing CD163 expression after rWb123 incubation in uPAR antibody pre-treated and untreated cells. Intensity was determined from cells (Violin plot, n = 90, *represents significance values compared to control, ****p < 0.0001). Statistical significance was calculated using unpaired t test. **B)** Blots showing pSTAT3 and STAT3 expression in rWb123 treated cells compared to control cells, across varying rWb123 concentrations. The concentrations are denoted as +(125 ng), ++(250 ng), +++(500 ng), and ++++(1 µg). **C)** Blots showing CD163 expression in rWb123 treated cells compared with cells pre-treated with SN50i and TIRAPi.
(TIF)

S6 Fig Filarial serpin Wb123 attenuates the LPS and IFN-γ induced responses. **A)** Images showing CD163 expression in control cells compared with cells treated with LPS-I alone and LPS-I with rWb123. Intensity was determined from cells (Mean ± SEM, n = 70, *p = 0.0132, ****p < 0.0001), Statistical significance was calculated using ordinary one-way ANOVA. **B)** Images showing NO expression in LPS-I treated cells compared to LPS-I with rWb123 treated cells. Intensity was determined from cells (Mean ± SEM, n = 40, ****p < 0.0001), Statistical significance was calculated using ordinary one-way ANOVA. **C)** Immunofluorescence images showing uPA expression in control cells compared with LPS-I alone and LPS-I along with rWb123. Intensity was determined from cells (Mean ± SEM, n = 40, **p = 0.0025, ****p < 0.0001), Statistical significance was calculated using ordinary one-way ANOVA. **D)** Graph showing anti-Wb123 monoclonal antibody MabG8 binding with recombinant Wb123 using ELISA.
(TIF)

S1 Text **Table A.** Superimposed structure of Wb123 (Green) with resolved structure of *Homo sapiens* serpins (Cyan). **Table B.** Antibody details. **Table C.** Primer details.
(DOCX)

S1 Data Spreadsheet with data points for immunofluorescence assay, qPCR and uPA inhibition assay. (XLSX)

S2 Data Full images of western blot experiments. (PDF)

## References

[1] Cross JH. Filarial Nematodes. In: Baron S, editor. Medical Microbiology [Internet]. 4th ed. Galveston (TX): University of Texas Medical Branch at Galveston; 1996 [cited 2025 Jan 29]. Available from: http://www.ncbi.nlm.nih.gov/books/NBK7844/21413271

[2] Lymphatic filariasis [Internet]. [cited 2025 Jan 19]. Available from: https://www.who.int/news-room/fact-sheets/detail/lymphatic-filariasis

[3] Shrivastava A, Arora P, Khare A, Goel G, Kapoor N. Central nervous system filariasis masquerading as a glioma: case report. J Neurosurg. 2017;127(3):691–3. doi: 10.3171/2016.9.JNS161092 28009239

[4] Berkowitz AL, Raibagkar P, Pritt BS, Mateen FJ. Neurologic manifestations of the neglected tropical diseases. J Neurol Sci. 2015;349(1–2):20–32. doi: 10.1016/j.jns.2015.01.001 25623803

[5] O’Regan NL, Steinfelder S, Venugopal G, Rao GB, Lucius R, Srikantam A, et al. Brugia malayi microfilariae induce a regulatory monocyte/macrophage phenotype that suppresses innate and adaptive immune responses. PLoS Negl Trop Dis. 2014;8(10):e3206. doi: 10.1371/journal.pntd.0003206 25275395 PMC4183501

[6] Semnani RT, Liu AY, Sabzevari H, Kubofcik J, Zhou J, Gilden JK, et al. Brugia malayi microfilariae induce cell death in human dendritic cells, inhibit their ability to make IL-12 and IL-10, and reduce their capacity to activate CD4+ T cells. J Immunol. 2003;171(4):1950–60. doi: 10.4049/jimmunol.171.4.1950 12902498

[7] Semnani RT, Law M, Kubofcik J, Nutman TB. Filaria-induced immune evasion: suppression by the infective stage of Brugia malayi at the earliest host-parasite interface. J Immunol. 2004;172(10):6229–38. doi: 10.4049/jimmunol.172.10.6229 15128811

[8] Wink DA, Hines HB, Cheng RYS, Switzer CH, Flores-Santana W, Vitek MP. Nitric oxide and redox mechanisms in the immune response. J Leukoc Biol. 2011;89(6):873–91. 21233414 10.1189/jlb.1010550 PMC3100761

[9] Orecchioni M, Ghosheh Y, Pramod AB, Ley K. Macrophage polarization: different gene signatures in M1 (LPS) vs. classically and M2 (LPS–) vs. alternatively activated macrophages. Front Immunol. 2019. doi: 10.3389/fimmu.2019.01084 PMC6543837 31178859

[10] Alternatively Activated and Immunoregulatory Monocytes in Human Filarial Infections. The Journal of Infectious Diseases | Oxford Academic [Internet]. [cited 2024 Oct 25]. Available from: https://academic.oup.com/jid/article/199/12/1827/881801?login=false 10.1086/599090 PMC3440875 19456233

[11] Mukherjee S, Mukherjee S, Maiti TK, Bhattacharya S, Sinha Babu SP. A Novel Ligand of Toll-like Receptor 4 From the Sheath of Wuchereria bancrofti Microfilaria Induces Proinflammatory Response in Macrophages. J Infect Dis. 2017;215(6):954–65. doi: 10.1093/infdis/jix067 28453850

[12] Mukherjee S u, Mukherjee S a, Bhattacharya S, Sinha Babu SP. Surface proteins of Setaria cervi induce inflammation in macrophage through Toll-like receptor 4 (TLR4)-mediated signalling pathway. Parasite Immunol. 2017;39(1):e12389. 10.1111/pim.12389 27659561

[13] Weinkopff T, Mackenzie C, Eversole R, Lammie PJ. Filarial excretory-secretory products induce human monocytes to produce lymphangiogenic mediators. PLoS Negl Trop Dis. 2014;8(7):e2893. doi: 10.1371/journal.pntd.0002893 25010672 PMC4091784

[14] Semnani RT, Keiser PB, Coulibaly YI, Keita F, Diallo AA, Traore D, et al. Filaria-induced monocyte dysfunction and its reversal following treatment. Infect Immun. 2006;74(8):4409–17. doi: 10.1128/IAI.01106-05 16861626 PMC1539612

[15] Kaushal NA, Hussain R, Nash TE, Ottesen EA. Identification and characterization of excretory-secretory products of Brugia malayi, adult filarial parasites. J Immunol. 1982;129(1):338–43. doi: 10.4049/jimmunol.129.1.338 7086135

[16] Moreno Y, Geary TG. Stage- and gender-specific proteomic analysis of Brugia malayi excretory-secretory products. PLOS Negl Trop Dis. 2008;2(10):e326. doi: 10.1371/journal.pntd.0000326 PMC2569413 18958170

[17] Khan MS, Singh P, Azhar A, Naseem A, Rashid Q, Kabir MA, et al. Serpin Inhibition Mechanism: A Delicate Balance between Native Metastable State and Polymerization. J Amino Acids. 2011;2011:606797. doi: 10.4061/2011/606797 22312466 PMC3268027

[18] Sanrattana W, Maas C, de Maat S. SERPINs—From Trap to Treatment. Front Med [Internet]. 2019 [cited 2024 Oct 19];6. Available from: https://www.frontiersin.org/journals/medicine/articles/10.3389/fmed.2019.00025/full 10.3389/fmed.2019.00025 PMC6379291 30809526

[19] Xu N, Bai X, Liu Y, Yang Y, Tang B, Shi HN, et al. The Anti-Inflammatory Immune Response in Early Trichinella spiralis Intestinal Infection Depends on Serine Protease Inhibitor-Mediated Alternative Activation of Macrophages. J Immunol. 2021;206(5):963–77. doi: 10.4049/jimmunol.2000290 33495238 PMC7887736

[20] Madeira F, Madhusoodanan N, Lee J, Eusebi A, Niewielska A, Tivey ARN, et al. The EMBL-EBI Job Dispatcher sequence analysis tools framework in 2024. Nucleic Acids Research. 2024;52(W1):W521-5. 10.1093/nar/gkae241 PMC11223882 38597606

[21] Bennuru S, Semnani R, Meng Z, Ribeiro JMC, Veenstra TD, Nutman TB. Brugia malayi excreted/secreted proteins at the host/parasite interface: stage- and gender-specific proteomic profiling. PLOS Negl Trop Dis. 2009;3(4):e410. doi: 10.1371/journal.pntd.0000410 PMC2659452 19352421

[22] Hamada M, Varkoly KS, Riyadh O, Beladi R, Munuswamy-Ramanujam G, Rawls A, et al. Urokinase-Type Plasminogen Activator Receptor (uPAR) in Inflammation and Disease: A Unique Inflammatory Pathway Activator. Biomedicines. 2024;12(6):1167. doi: 10.3390/biomedicines12061167 38927374 PMC11201033

[23] Heissig B, Salama Y, Takahashi S, Osada T, Hattori K. The multifaceted role of plasminogen in inflammation. Cell Signal. 2020;75:109761. doi: 10.1016/j.cellsig.2020.109761 32861744 PMC7452830

[24] Vago JP, Sugimoto MA, Lima KM, Negreiros-Lima GL, Baik N, Teixeira MM. Plasminogen and the plasminogen receptor, Plg-RKT, regulate macrophage phenotypic, and functional changes. Front Immunol. 2019;10:1458. 31316511 10.3389/fimmu.2019.01458 PMC6611080

[25] Serine Protease, Enzyme Catalysis | Learn Science at Scitable [Internet]. [cited 2025 Jan 19]. Available from: http://www.nature.com/scitable/topicpage/enzyme-catalysis-the-serine-proteases-14398894

[26] Mangan MSJ, Kaiserman D, Bird PI. The role of serpins in vertebrate immunity. Tissue Antigens. 2008;72(1):1–10. doi: 10.1111/j.1399-0039.2008.01059.x 18498291

[27] Etzerodt A, Moestrup SK. CD163 and inflammation: biological, diagnostic, and therapeutic aspects. Antioxid Redox Signal. 2013;18(17):2352–63. doi: 10.1089/ars.2012.4834 22900885 PMC3638564

[28] Willment JA, Lin HH, Reid DM, Taylor PR, Williams DL, Wong SYC, et al. Dectin-1 expression and function are enhanced on alternatively activated and GM-CSF-treated macrophages and are negatively regulated by IL-10, dexamethasone, and lipopolysaccharide. J Immunol. 2003;171(9):4569–73. 14568930 10.4049/jimmunol.171.9.4569

[29] Taddio MF, Castro Jaramillo CA, Runge P, Blanc A, Keller C, Talip Z, et al. In Vivo Imaging of Local Inflammation: Monitoring LPS-Induced CD80/CD86 Upregulation by PET. Mol Imaging Biol. 2021;23(2):196–207. doi: 10.1007/s11307-020-01543-3 32989622 PMC7910267

[30] Bhattacharjee A, Shukla M, Yakubenko VP, Mulya A, Kundu S, Cathcart MK. IL-4 and IL-13 employ discrete signaling pathways for target gene expression in alternatively activated monocytes/macrophages. Free Radic Biol Med. 2013;54:1–16. doi: 10.1016/j.freeradbiomed.2012.10.553 23124025 PMC3534796

[31] IL-10/STAT3-mediated anti-inflammatory response: recent developments and future challenges | Briefings in Functional Genomics | Oxford Academic [Internet]. [cited 2025 Jan 19]. Available from: https://academic.oup.com/bfg/article/12/6/489/238388 10.1093/bfgp/elt028 PMC3838198 23943603

[32] Kubala MH, Punj V, Placencio-Hickok VR, Fang H, Fernandez GE, Sposto R, et al. Plasminogen Activator Inhibitor-1 Promotes the Recruitment and Polarization of Macrophages in Cancer. Cell Rep. 2018;25(8):2177-2191.e7. doi: 10.1016/j.celrep.2018.10.082 30463014 PMC6876299

[33] Xing Z, Gauldie J, Cox G, Baumann H, Jordana M, Lei XF, et al. IL-6 is an antiinflammatory cytokine required for controlling local or systemic acute inflammatory responses. J Clin Invest. 1998;101(2):311–20. doi: 10.1172/JCI1368 9435302 PMC508569

[34] Hsu P-C, Chen Y-H, Cheng C-F, Kuo C-Y, Sytwu H-K. Interleukin-6 and Interleukin-8 Regulate STAT3 Activation Migration/Invasion and EMT in Chrysophanol-Treated Oral Cancer Cell Lines. Life (Basel). 2021;11(5):423. doi: 10.3390/life11050423 34063134 PMC8148210

[35] Wu J, Gao F-X, Wang C, Qin M, Han F, Xu T, et al. IL-6 and IL-8 secreted by tumour cells impair the function of NK cells via the STAT3 pathway in oesophageal squamous cell carcinoma. J Exp Clin Cancer Res. 2019;38(1):321. doi: 10.1186/s13046-019-1310-0 31324197 PMC6642486

[36] Herbein G, Varin A. The macrophage in HIV-1 infection: from activation to deactivation? Retrovirology. 2010;7:33. doi: 10.1186/1742-4690-7-33 20380696 PMC2859752

[37] Munan S, Mondal A, Shailja S, Pati S, Samanta A. Unique synthetic strategy for probing in situ lysosomal NO for screening neuroinflammatory phenotypes against SARS-CoV-2 RNA in phagocytotic microglia. Anal Chem. 2024;96(19):7479–86. 38689560 10.1021/acs.analchem.3c05981

[38] Fuhrman B, Gantman A, Khateeb J, Volkova N, Horke S, Kiyan J, et al. Urokinase activates macrophage PON2 gene transcription via the PI3K/ROS/MEK/SREBP-2 signalling cascade mediated by the PDGFR-beta. Cardiovasc Res. 2009;84(1):145–54. doi: 10.1093/cvr/cvp184 19497963

[39] Myxoma viral serpin, Serp-1, inhibits human monocyte adhesion through regulation of actin-binding protein filamin B | Journal of Leukocyte Biology | Oxford Academic [Internet]. [cited 2025 Apr 7]. Available from: https://academic.oup.com/jleukbio/article/85/3/418/6974918 10.1189/jlb.0808506 19052145

[40] Kwiecień I, Polubiec-Kownacka M, Dziedzic D, Wołosz D, Rzepecki P, Domagała-Kulawik J. CD163 and CCR7 as markers for macrophage polarization in lung cancer microenvironment. Cent Eur J Immunol. 2019;44(4):395–402. doi: 10.5114/ceji.2019.92795 32140052 PMC7050058

[41] Fischer-Riepe L, Daber N, Schulte-Schrepping J, Véras De Carvalho BC, Russo A, Pohlen M, et al. CD163 expression defines specific, IRF8-dependent, immune-modulatory macrophages in the bone marrow. J Allergy Clin Immunol. 2020;146(5):1137–51. doi: 10.1016/j.jaci.2020.02.034 32199911

[42] Stempin CC, Dulgerian LR, Garrido VV, Cerban FM. Arginase in Parasitic Infections: Macrophage Activation, Immunosuppression, and Intracellular Signals. J Biomed Biotechnol. 2010;2010:683485. 20029630 10.1155/2010/683485 PMC2792949

[43] Canè S, Geiger R, Bronte V. The roles of arginases and arginine in immunity. Nat Rev Immunol. 2025;25(4):266–84. doi: 10.1038/s41577-024-01098-2 39420221

[44] Strizova Z, Benesova I, Bartolini R, Novysedlak R, Cecrdlova E, Foley LK, et al. M1/M2 macrophages and their overlaps - myth or reality? Clin Sci (Lond). 2023;137(15):1067–93. doi: 10.1042/CS20220531 37530555 PMC10407193

[45] Al Madhoun A, Kochumon S, Al-Rashed F, Sindhu S, Thomas R, Miranda L, et al. Dectin-1 as a Potential Inflammatory Biomarker for Metabolic Inflammation in Adipose Tissue of Individuals with Obesity. Cells. 2022;11(18):2879. doi: 10.3390/cells11182879 36139454 PMC9496833

[46] Preissner KT, Kanse SM, May AE. Urokinase receptor: a molecular organizer in cellular communication. Curr Opin Cell Biol. 2000;12(5):621–8. doi: 10.1016/s0955-0674(00)00141-1 10978899

[47] Farias-Eisner R, Vician L, Silver A, Reddy S, Rabbani SA, Herschman HR. The urokinase plasminogen activator receptor (UPAR) is preferentially induced by nerve growth factor in PC12 pheochromocytoma cells and is required for NGF-driven differentiation. J Neurosci. 2000;20(1):230–9. 10627600 10.1523/JNEUROSCI.20-01-00230.2000 PMC6774117

[48] Tang L, Han X. The urokinase plasminogen activator system in breast cancer invasion and metastasis. Biomed Pharmacother. 2013;67(2):179–82. doi: 10.1016/j.biopha.2012.10.003 23201006

[49] Zhai B-T, Tian H, Sun J, Zou J-B, Zhang X-F, Cheng J-X, et al. Urokinase-type plasminogen activator receptor (uPAR) as a therapeutic target in cancer. J Transl Med. 2022;20(1):135. doi: 10.1186/s12967-022-03329-3 35303878 PMC8932206

[50] Blasi F, Carmeliet P. uPAR: a versatile signalling orchestrator. Nat Rev Mol Cell Biol. 2002;3(12):932–43. doi: 10.1038/nrm977 12461559

[51] Smith HW, Marshall CJ. Regulation of cell signalling by uPAR. Nat Rev Mol Cell Biol. 2010;11(1):23–36. doi: 10.1038/nrm2821 20027185

[52] Chen J, Baskerville C, Han Q, Pan ZK, Huang S. Alpha(v) integrin, p38 mitogen-activated protein kinase, and urokinase plasminogen activator are functionally linked in invasive breast cancer cells. J Biol Chem. 2001;276(51):47901–5. doi: 10.1074/jbc.M107574200 11606583

[53] Genua M, D’Alessio S, Cibella J, Gandelli A, Sala E, Correale C, et al. The urokinase plasminogen activator receptor (uPAR) controls macrophage phagocytosis in intestinal inflammation. 2015 [cited 2025 Apr 14]; Available from: https://gut.bmj.com/content/64/4/589.long 10.1136/gutjnl-2013-305933 24848264

[54] Mauer J, Chaurasia B, Goldau J, Vogt MC, Ruud J, Nguyen KD, et al. Signaling by IL-6 promotes alternative activation of macrophages to limit endotoxemia and obesity-associated resistance to insulin. Nat Immunol. 2014;15(5):423–30. doi: 10.1038/ni.2865 24681566 PMC4161471

[55] Ackermann J, Arndt L, Fröba J, Lindhorst A, Glaß M, Kirstein M, et al. IL-6 signaling drives self-renewal and alternative activation of adipose tissue macrophages. Front Immunol. 2024;15:1201439. doi: 10.3389/fimmu.2024.1201439 38482013 PMC10933059

[56] Fuster JJ, Walsh K. The good, the bad, and the ugly of interleukin-6 signaling. EMBO J. 2014;33(13):1425–7. 24850773 10.15252/embj.201488856 PMC4194086

[57] Marella S, Sharma A, Ganesan V, Ferrer-Torres D, Krempski JW, Idelman G, et al. IL-13-induced STAT3-dependent signaling networks regulate esophageal epithelial proliferation in eosinophilic esophagitis. J Allergy Clin Immunol. 2023;152(6):1550–68. doi: 10.1016/j.jaci.2023.07.021 37652141 PMC11102758

[58] Rahaman SO, Vogelbaum MA, Haque SJ. Aberrant Stat3 signaling by interleukin-4 in malignant glioma cells: involvement of IL-13Ralpha2. Cancer Res. 2005;65(7):2956–63. doi: 10.1158/0008-5472.CAN-04-3592 15805299

[59] Hutchins AP, Diez D, Miranda-Saavedra D. The IL-10/STAT3-mediated anti-inflammatory response: recent developments and future challenges. Brief Funct Genomics. 2013;12(6):489–98. doi: 10.1093/bfgp/elt028 23943603 PMC3838198

[60] Takeda K, Clausen BE, Kaisho T, Tsujimura T, Terada N, Förster I, et al. Enhanced Th1 activity and development of chronic enterocolitis in mice devoid of Stat3 in macrophages and neutrophils. Immunity. 1999;10(1):39–49. doi: 10.1016/s1074-7613(00)80005-9 10023769

[61] El Kasmi KC, Holst J, Coffre M, Mielke L, de Pauw A, Lhocine N, et al. General nature of the STAT3-activated anti-inflammatory response. J Immunol. 2006;177(11):7880–8. doi: 10.4049/jimmunol.177.11.7880 17114459

[62] Mosser DM. The many faces of macrophage activation. J Leukoc Biol. 2003;73(2):209–12. doi: 10.1189/jlb.0602325 12554797

[63] Chen R, Yang D, Shen L, Fang J, Khan R, Liu D. Overexpression of CD86 enhances the ability of THP-1 macrophages to defend against Talaromyces marneffei. Immun Inflamm Dis. 2022;10(12):e740. doi: 10.1002/iid3.740 36444627 PMC9673424

[64] Fang FC. Antimicrobial reactive oxygen and nitrogen species: concepts and controversies. Nat Rev Microbiol. 2004;2(10):820–32. doi: 10.1038/nrmicro1004 15378046

[65] Martinvalet D, Walch M. Editorial: The Role of Reactive Oxygen Species in Protective Immunity. Front Immunol [Internet]. 2022 [cited 2025 Apr 6];12. Available from: https://www.frontiersin.org/journals/immunology/articles/10.3389/fimmu.2021.832946/full 10.3389/fimmu.2021.832946 PMC8821872 35145515

[66] Müller E, Christopoulos PF, Halder S, Lunde A, Beraki K, Speth M. Toll-Like Receptor Ligands and Interferon-γ Synergize for Induction of Antitumor M1 Macrophages. Front Immunol. 2017;8. doi: 10.3389/fimmu.2017.01383 PMC5662546 29123526

[67] Rasmussen LJH, Petersen JEV, Eugen-Olsen J. Soluble Urokinase Plasminogen Activator Receptor (suPAR) as a Biomarker of Systemic Chronic Inflammation. Front Immunol. 2021;12:780641. doi: 10.3389/fimmu.2021.780641 34925360 PMC8674945

[68] Sharma A, Sharma P, Ganga L, Satoeya N, Mishra S, Vishwakarma AL, et al. Infective Larvae of Brugia malayi Induce Polarization of Host Macrophages that Helps in Immune Evasion. Front Immunol. 2018;9:194. doi: 10.3389/fimmu.2018.00194 29483912 PMC5816041

