# Supplementary figures and images for: Reversal of filarial serpin Wb123-urokinase plasminogen activator receptor mediated alternative macrophage activation by monoclonal antibody
Source: PLoS Negl Trop Dis. 2025 Dec 22;19(12):e0013726. doi: 10.1371/journal.pntd.0013726 (PMC12768378; doi:10.1371/journal.pntd.0013726)

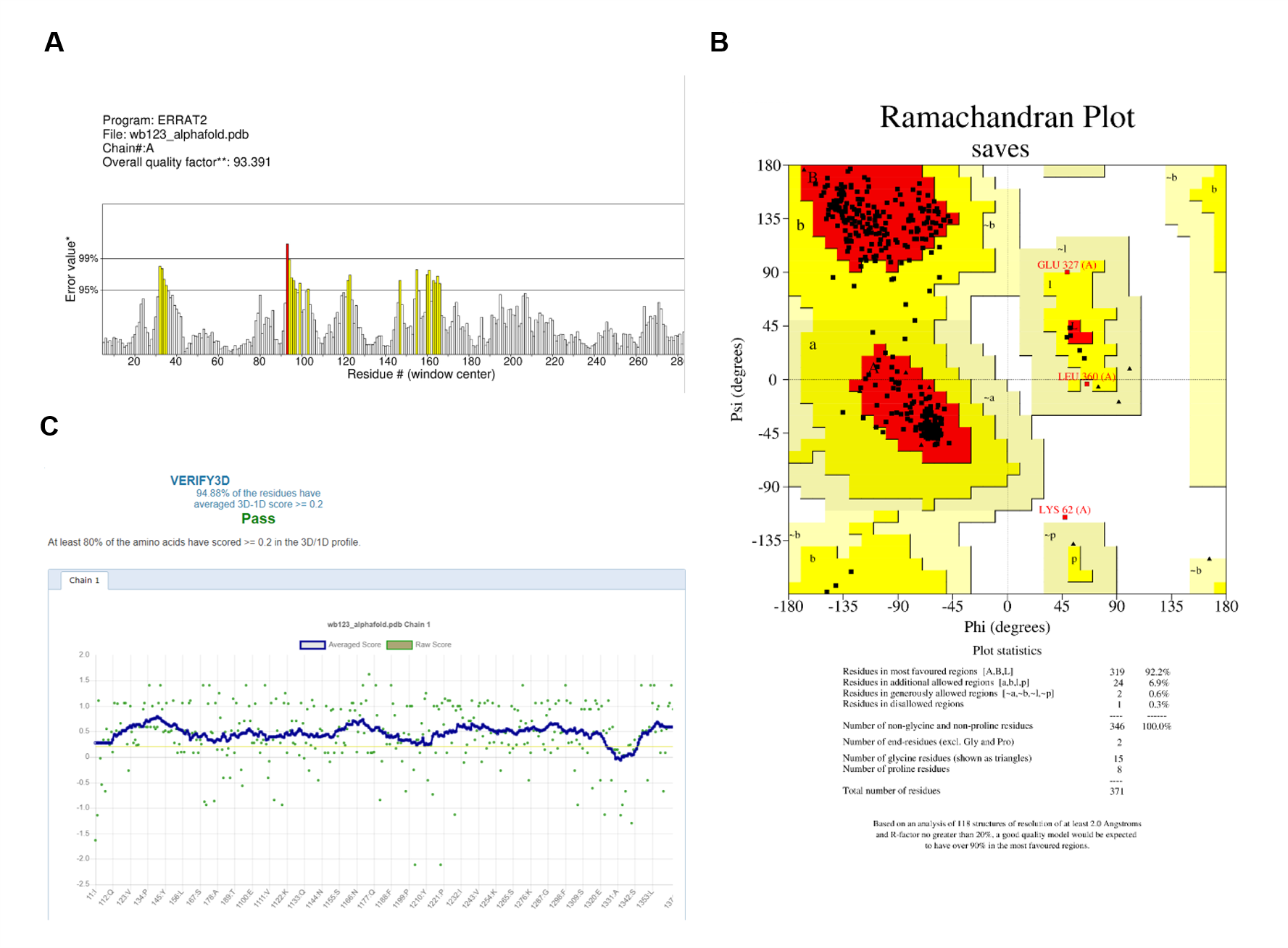

Supplement: S1 Fig — A) Graph showing Errat score of modelled Wb123 structure. B) Ramachandran plot showing residue analysis of Wb123 structure done using PROCHECK server. C) Graph showing verify-3D score of Wb123 structure. (TIF) [file pntd.0013726.s001.tif]

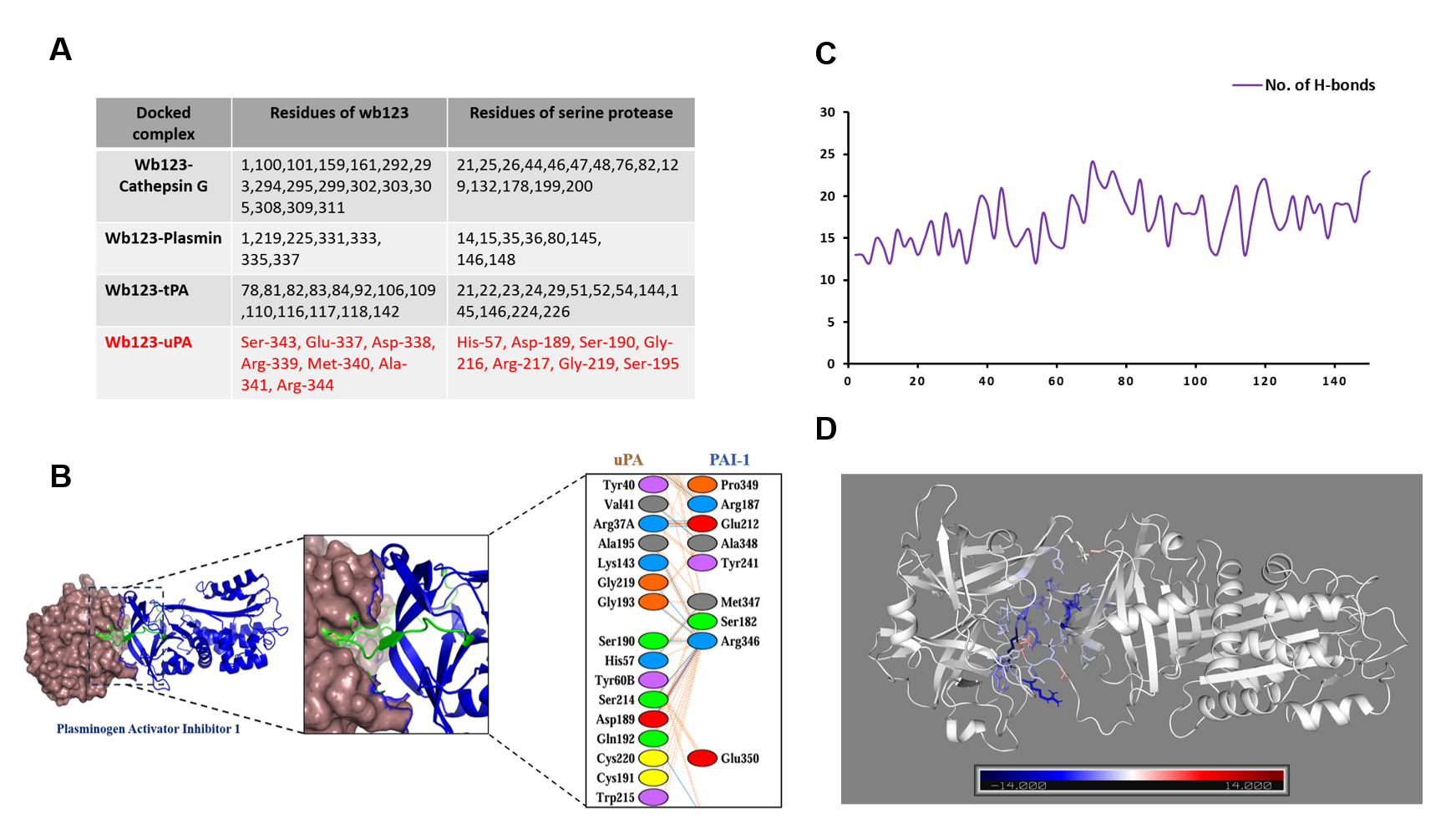

Supplement: S2 Fig — A) Table representing the amino acid residues involved in the interaction of Wb123 with different serine proteases. B) Biophysical interactions between human serpin PAI-1(shown in blue) and uPA (shown in brown). Zoom-in showing the binding interface between PAI-1 RCL residues and uPA catalytic site. C) Graph showing number of hydrogen bonds during 150ns simulation of Wb123-uPA complex. D) Three-dimensional representation of the uPA-Wb123 interface, with residues colored blue indicating favorable binding contributions and red indicating unfavorable contributions. (TIF) [file pntd.0013726.s002.tif]

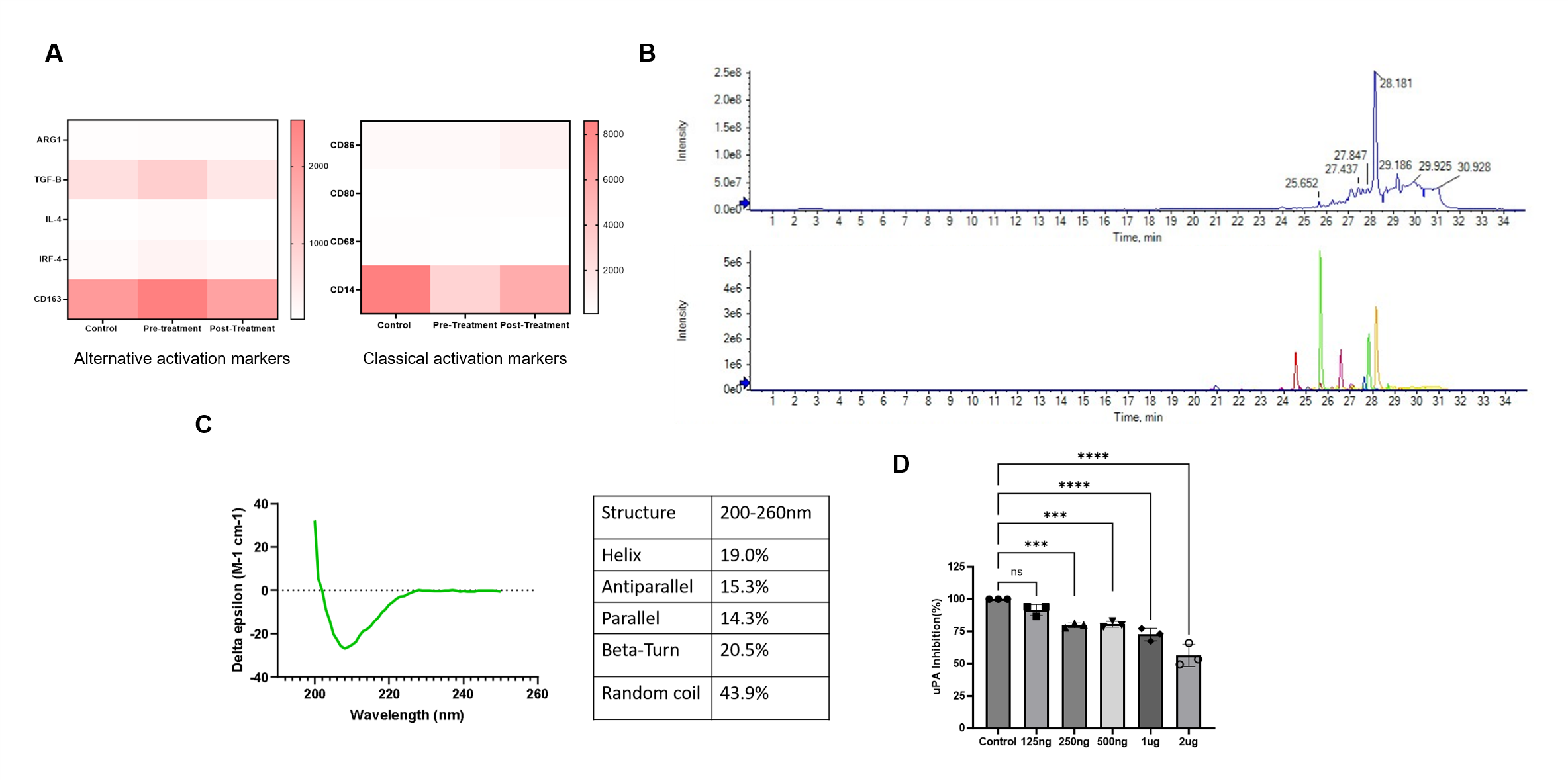

Supplement: S3 Fig — A) Heatmap represents expression of alternative and classical activation markers in pre-treatment, post-treatment and healthy person monocytes from gene expression browser. B) Image showing total ion chromatogram (TIC) and extracted ion chromatogram (XIC) of Wb123 from mass spectrometry analysis. C) Plot of circular dichroism spectra of Wb123 with secondary structure information. D) Bar graph showing uPA activity in varying rWb123 concentrations compared to control. (TIF) [file pntd.0013726.s003.tif]

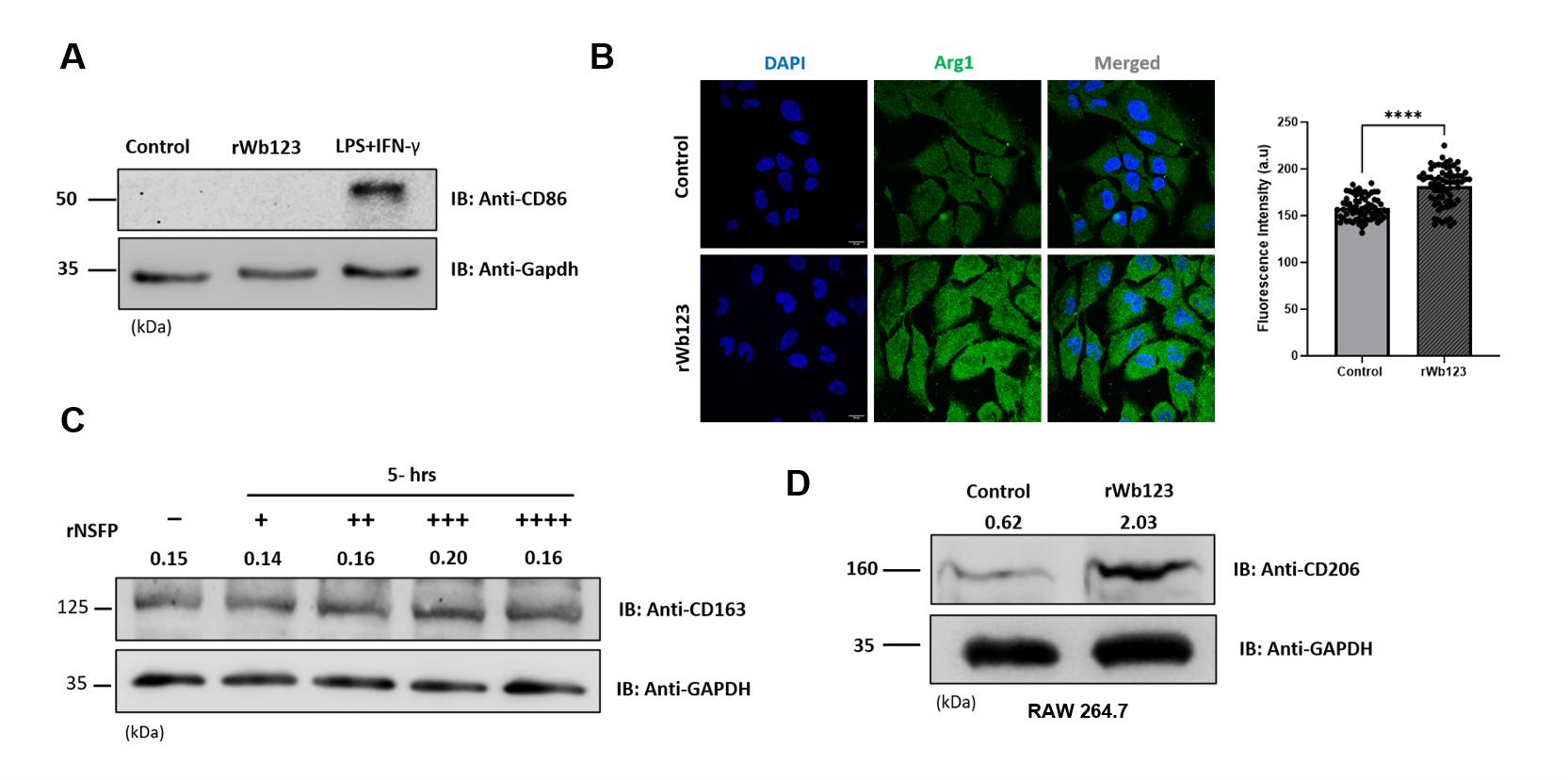

Supplement: S4 Fig — A) Blots showing CD86 expression in rWb123 treated cells, LPS-IFN-γ treated cells and control cells. B) Images showing Arg-1 expression in rWb123 treated cells and untreated cells. Intensity was determined from cells (Mean ± SEM, n = 70, *represents significance values compared to control, ****p < 0.0001), Statistical significance was calculated using unpaired t test. C) Blot showing CD163 expression in recombinant non-serpin filarial protein (rNSFP) treated cells, compared to control cells, across varying rNSFP concentrations. The concentrations are denoted as +(125 ng), ++(250 ng), +++(500 ng), and ++++(1 µg). CD163 expression levels are quantified as the CD163/Gapdh ratio. D) Blot showing CD206 expression in rWb123 treated mouse macrophages compared to control. (TIF) [file pntd.0013726.s004.tif]

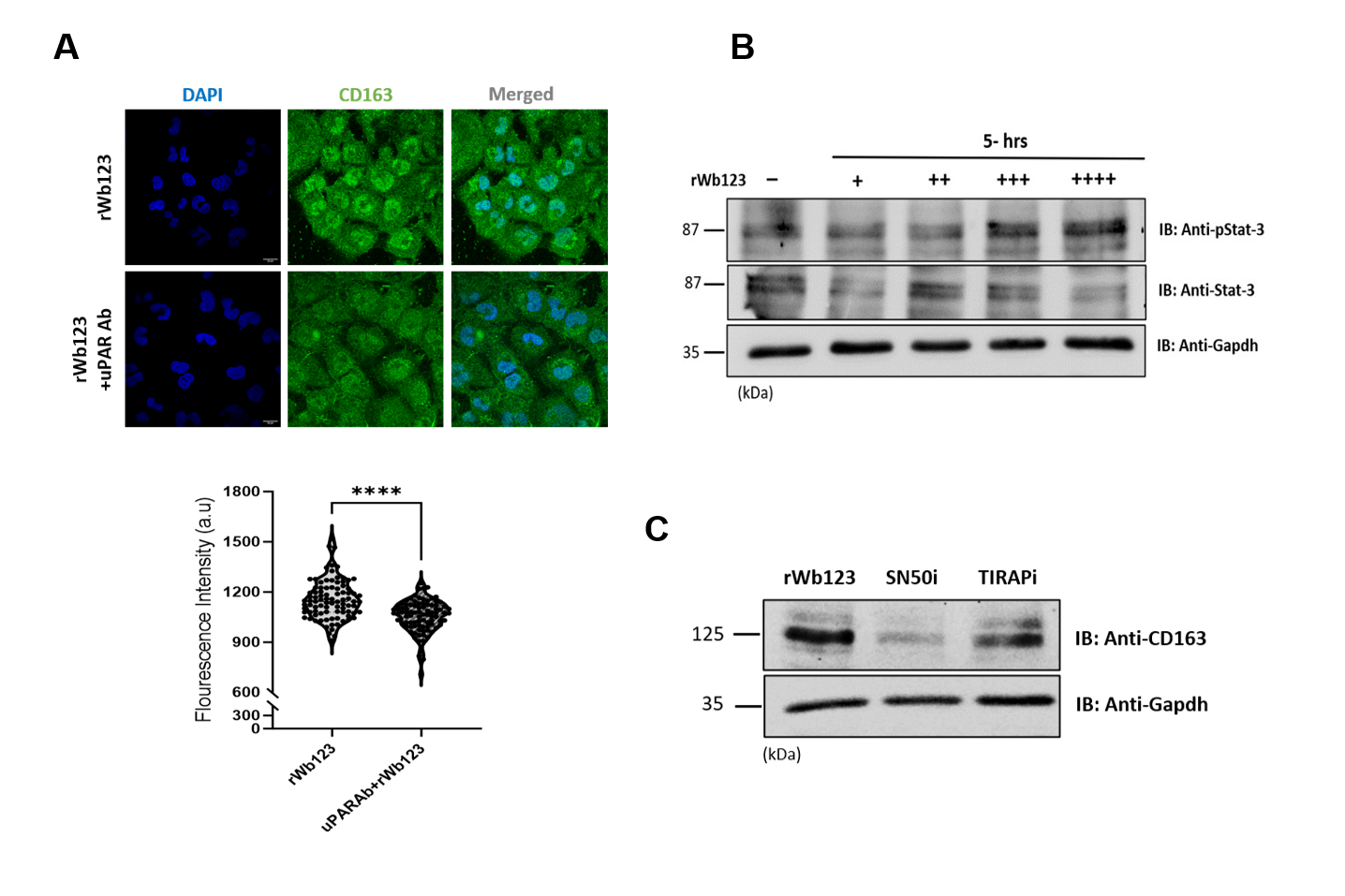

Supplement: S5 Fig — A) Images showing CD163 expression after rWb123 incubation in uPAR antibody pre-treated and untreated cells. Intensity was determined from cells (Violin plot, n = 90, *represents significance values compared to control, ****p < 0.0001). Statistical significance was calculated using unpaired t test. B) Blots showing pSTAT3 and STAT3 expression in rWb123 treated cells compared to control cells, across varying rWb123 concentrations. The concentrations are denoted as +(125 ng), ++(250 ng), +++(500 ng), and ++++(1 µg). C) Blots showing CD163 expression in rWb123 treated cells compared with cells pre-treated with SN50i and TIRAPi. (TIF) [file pntd.0013726.s005.tif]

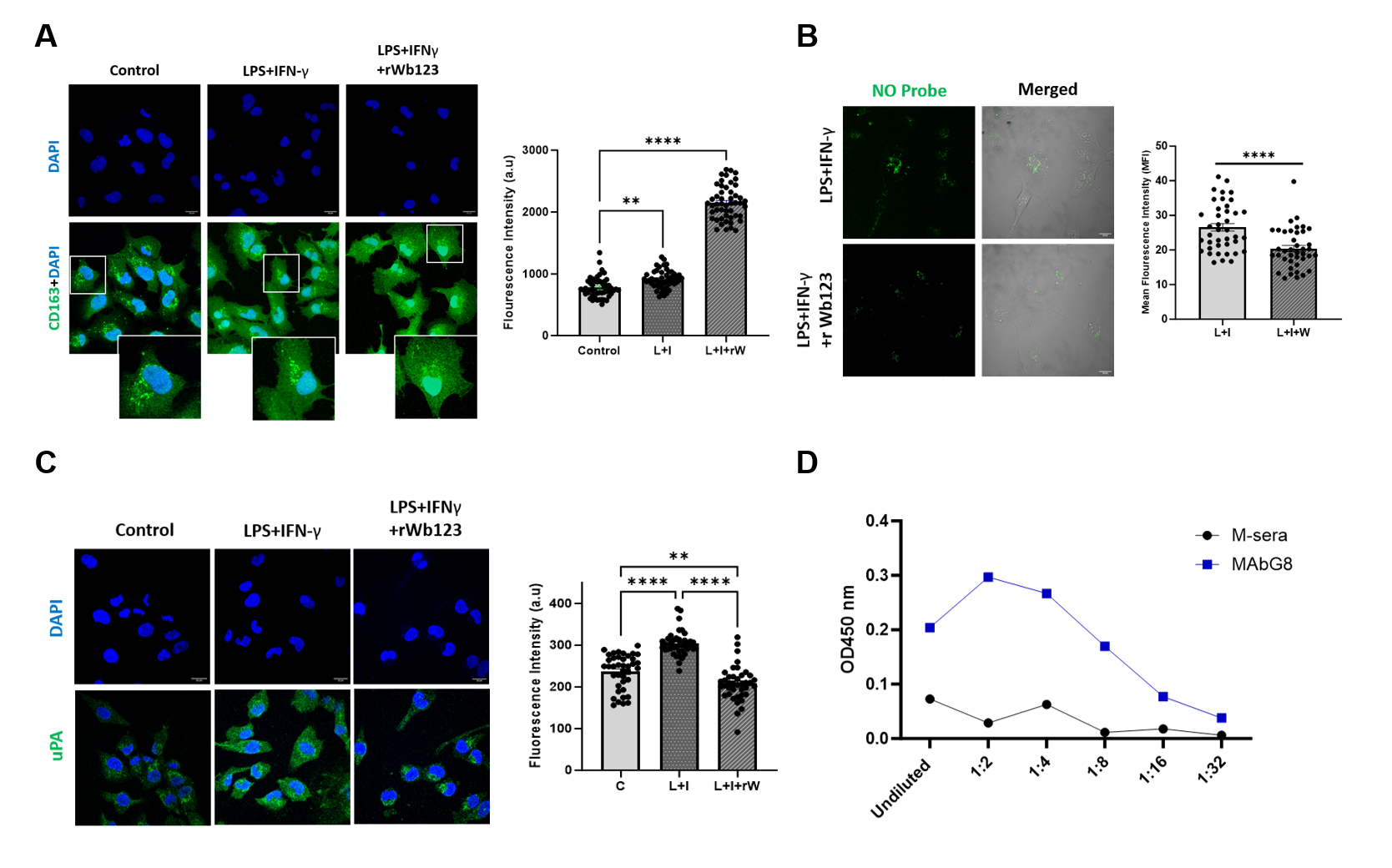

Supplement: S6 Fig — A) Images showing CD163 expression in control cells compared with cells treated with LPS-I alone and LPS-I with rWb123. Intensity was determined from cells (Mean ± SEM, n = 70, *p = 0.0132, ****p < 0.0001), Statistical significance was calculated using ordinary one-way ANOVA. B) Images showing NO expression in LPS-I treated cells compared to LPS-I with rWb123 treated cells. Intensity was determined from cells (Mean ± SEM, n = 40, ****p < 0.0001), Statistical significance was calculated using ordinary one-way ANOVA. C) Immunofluorescence images showing uPA expression in control cells compared with LPS-I alone and LPS-I along with rWb123. Intensity was determined from cells (Mean ± SEM, n = 40, **p = 0.0025, ****p < 0.0001), Statistical significance was calculated using ordinary one-way ANOVA. D) Graph showing anti-Wb123 monoclonal antibody MabG8 binding with recombinant Wb123 using ELISA. (TIF) [file pntd.0013726.s006.tif]
