# Supplementary material for: Reversal of filarial serpin Wb123-urokinase plasminogen activator receptor mediated alternative macrophage activation by monoclonal antibody
Source: PLoS Negl Trop Dis. 2025 Dec 22;19(12):e0013726. doi: 10.1371/journal.pntd.0013726 (PMC12768378; doi:10.1371/journal.pntd.0013726)
Supplement: S1 Text — Table A. Superimposed structure of Wb123 (Green) with resolved structure of Homo sapiens serpins (Cyan). Table B. Antibody details. Table C. Primer details. (DOCX) [file pntd.0013726.s007.docx]

**Supplementary Tables**

**Table A in S1 Text:** **Superimposed structure of Wb123 (Green) with resolved structure of *Homo sapiens* serpins (Cyan).**

|  | **Resolved Serpin Structures** | **PDB ID** | **RMSD (Å)** | **Superimposed Structure** |
| --- | --- | --- | --- | --- |
| Wb123  (Wuchereria bancrofti) | Plasminogen Activator Inhibitor-1 | 3PB1 | 1.530 | 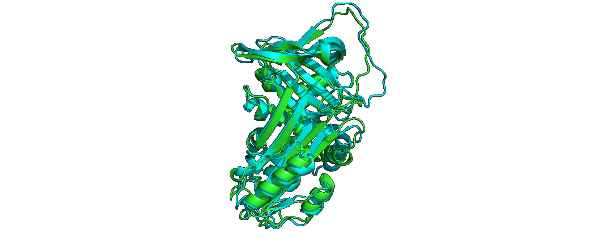 |
|  | Alpha-1-Antitrypsin | 1HP7 | 1.687 | 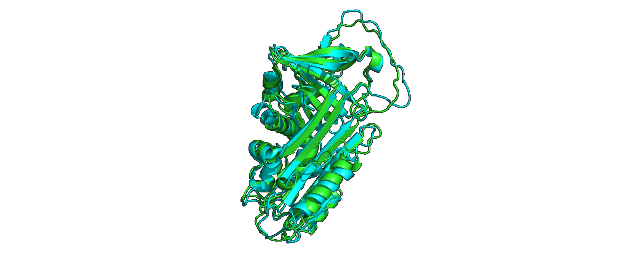 |
|  | Kallistatin | 6F02 | 1.845 | 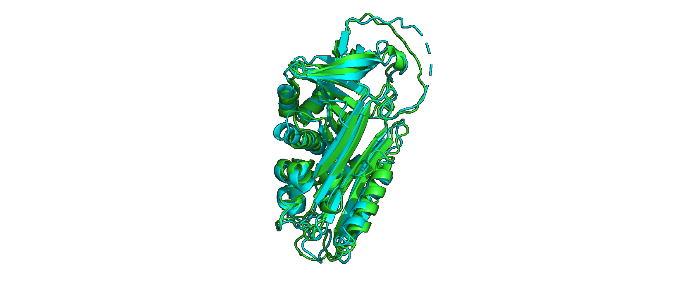 |
|  | MASPIN | 1XQP | 1.841 | 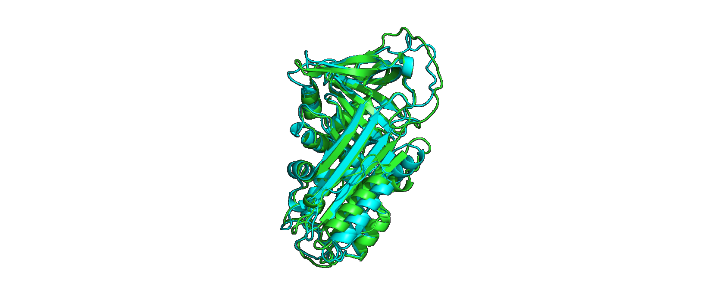 |
|  | Glia derived nexin | 4DY0 | 1.611 | 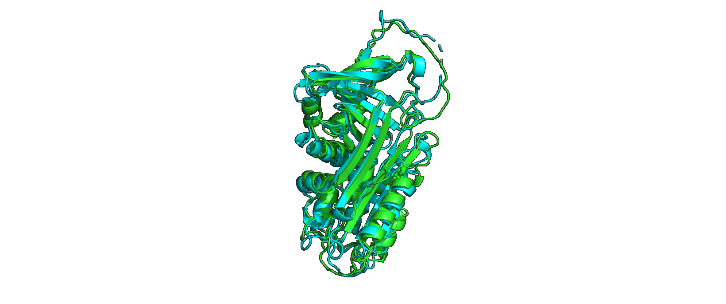 |
|  | Serpin B3 | 2ZV6 | 1.834 | 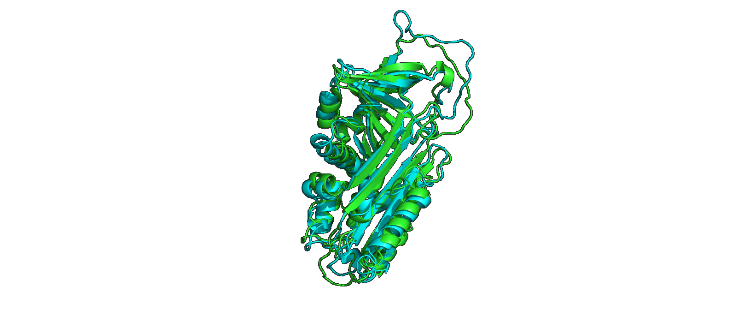 |

**Table B in S1 Text: Antibody details**

| **Antibodies** | **Identifier** |
| --- | --- |
| CD163 | Cat #DF8235 |
| CD206 | Cat #DF4149 |
| CD86 | Cat #DF6332 |
| STAT3 | Cat #AF6294 |
| PSTAT3 (Tyr705) | Cat #AF3293 |
| PLAU | Cat #DF6904 |
| ARG1 | Cat #DF6657 |
| PLAUR | Cat #DF12495 |
| MAbG8 (Wb123) | Tropical animal genetics Pvt. Ltd. |
| LRP1 | Cat #DF2935 |
| GAPDH | Cat #AF0911 |
| Anti-His | Cat # pAb-T0051 |
| Anti-rabbit HRP 2° | Cat #31460 |
| Anti-mouse HRP 2° | Cat #31430 |
| Alexa Fluor-488 | Cat #5196-2404 |

**Table C in S1 Text: Primer details**

| **Gene** | **Primers** |
| --- | --- |
| CD163_FP | CCAGAAGGAACTTGTAGCCACAG |
| CD163_RP | CAGGCACCAAGCGTTTTGAGCT |
| Dectin1_FP | ACAATGCTGGCAACTGGGCTCT |
| Dectin1_RP | AGAGCCATGGTACCTCAGTCTG |
| IL-4_FP | CCGTAACAGACATCTTTGCTGCC |
| IL-4_RP | GAGTGTCCTTCTCATGGTGGCT |
| IL-6_FP | AGACAGCCACTCACCTCTTCAG |
| IL-6_RP | TTCTGCCAGTGCCTCTTTGCTG |
| IL-8_FP | GAGAGTGATTGAGAGTGGACCAC |
| IL-8_RP | CACAACCCTCTGCACCCAGTTT |
| IL-10_FP | TCTCCGAGATGCCTTCAGCAGA |
| IL-10_RP | TCAGACAAGGCTTGGCAACCCA |
| Nf-kB_FP | GCACCCTGACCTTGCCTATT |
| Nf-kB_RP | GGTCCATCTCCTTGGTCTGC |
| 18S_FP | ACCCGTTGAACCCCATTCGTGA |
| 18S_RP | GCCTCACTAAACCATCCAATCGG |
