# Supplementary material for: Reversal of filarial serpin Wb123-urokinase plasminogen activator receptor mediated alternative macrophage activation by monoclonal antibody
Source: PLoS Negl Trop Dis. 2025 Dec 22;19(12):e0013726. doi: 10.1371/journal.pntd.0013726 (PMC12768378; doi:10.1371/journal.pntd.0013726)
Supplement: S2 Data — (PDF) [file pntd.0013726.s009.pdf]

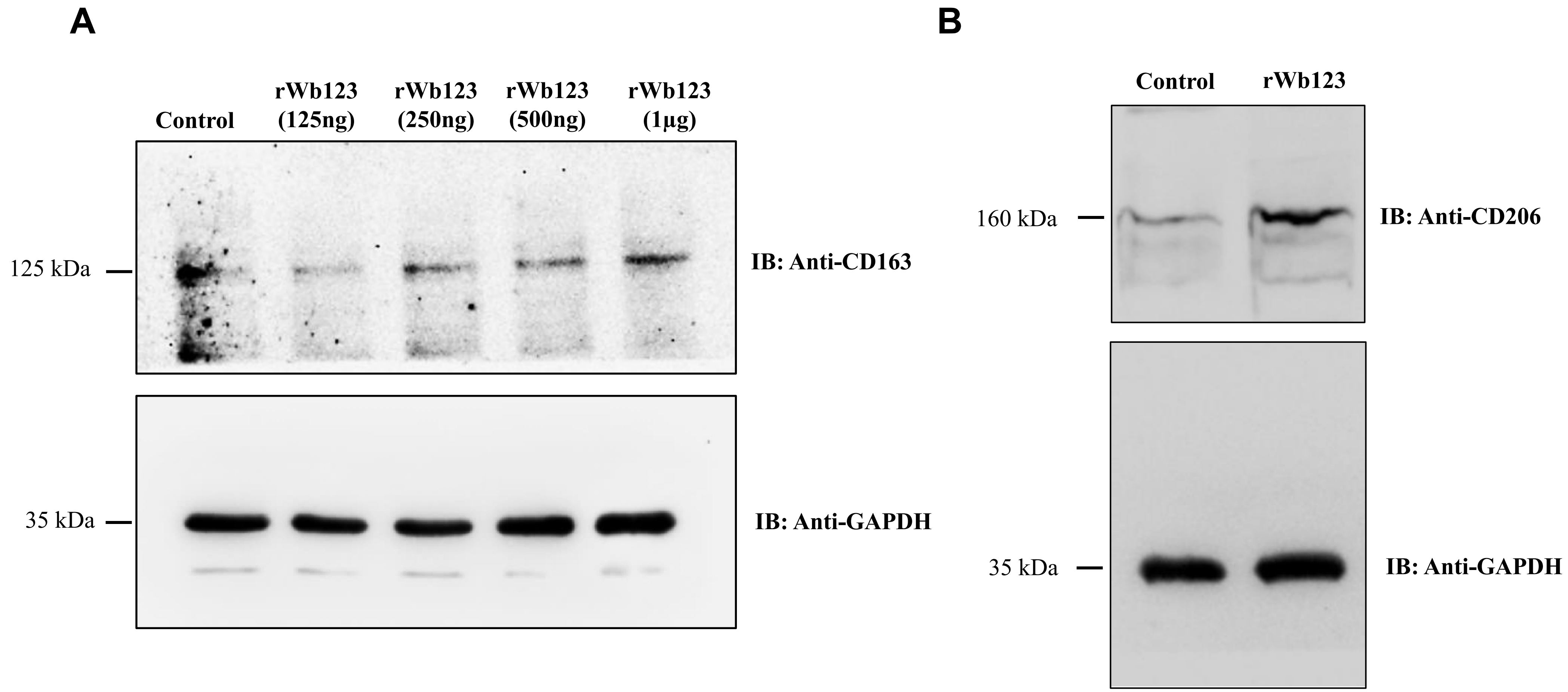

rWb123	CD163_Area	GAPDH_Area	Normalised Densitometry
**125ng**	112690	4460367	0.025264737
**250ng**	117334	3384369	0.034669387
**500ng**	204475	3017443	0.067764329
**750ng**	194860	4559551	0.042736664
**1µg**	285557	4669707	0.061150946

Sample	CD206_Area	GAPDH_Area	Normalised Densitometry
**Control**	1326798	2125913	0.624107384
**rWb123**	5183220	2552208	2.030876794

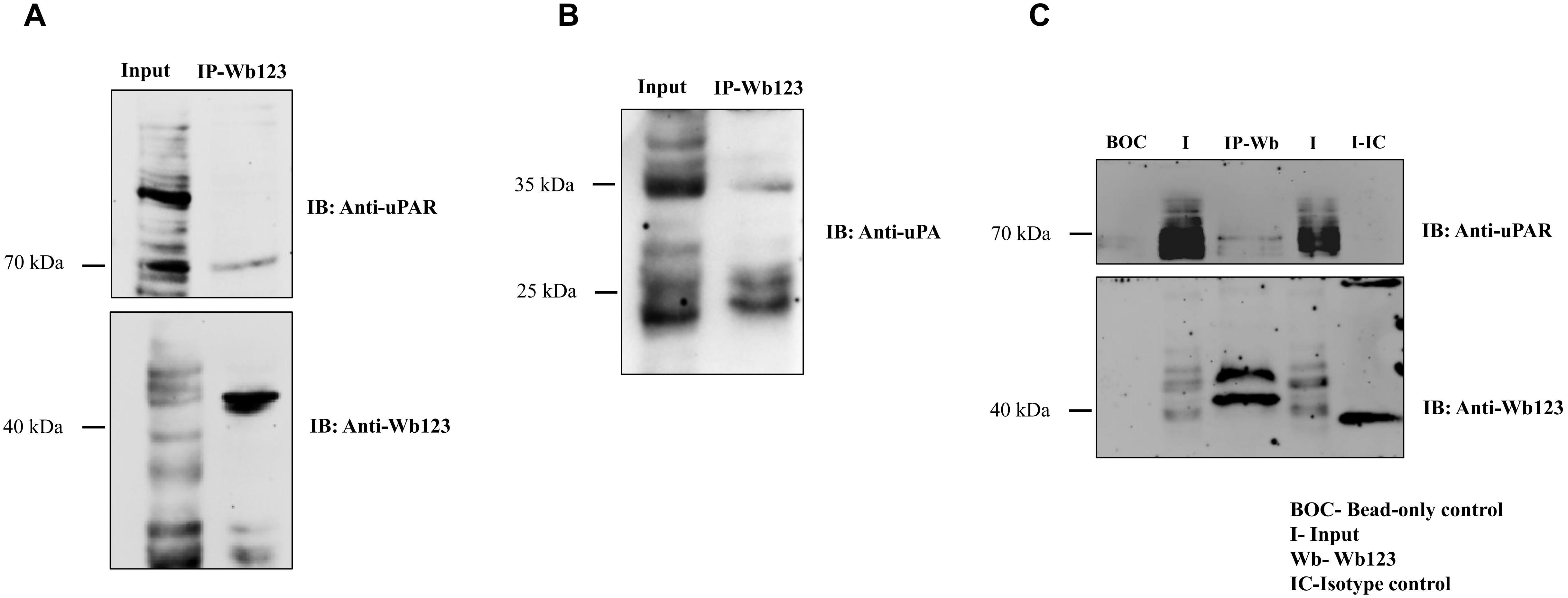
A
Input
IP-Wb123
IB: Anti-uPAR
70 kDa
IB: Anti-Wb123
40 kDa
B
Input
IP-Wb123
35 kDa
IB: Anti-uPA
25 kDa
C
BOC
I
IP-Wb
I
I-IC
70 kDa
IB: Anti-uPAR
IB: Anti-Wb123
40 kDa
BOC- Bead-only control
I- Input
Wb- Wb123
IC-Isotype control

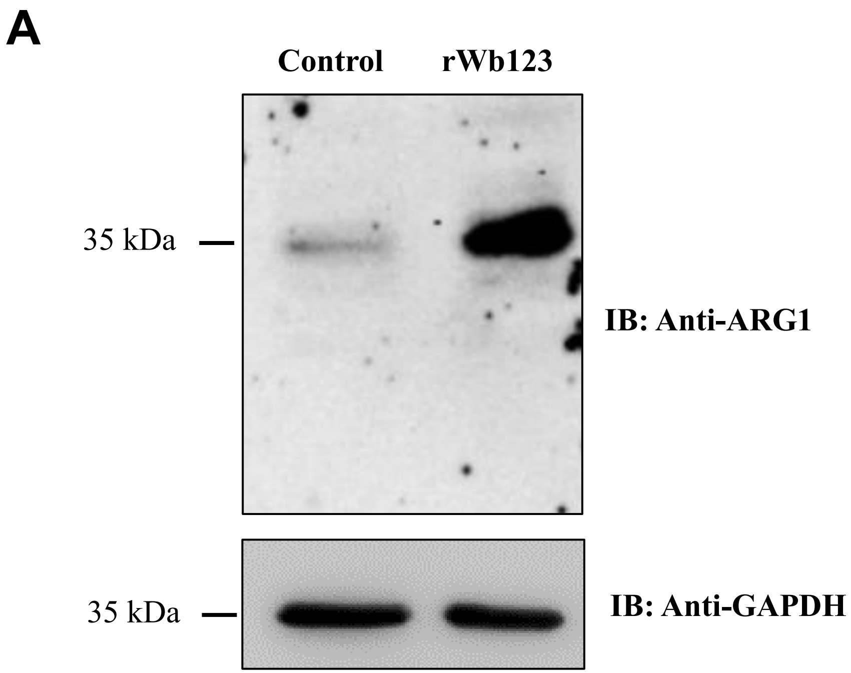

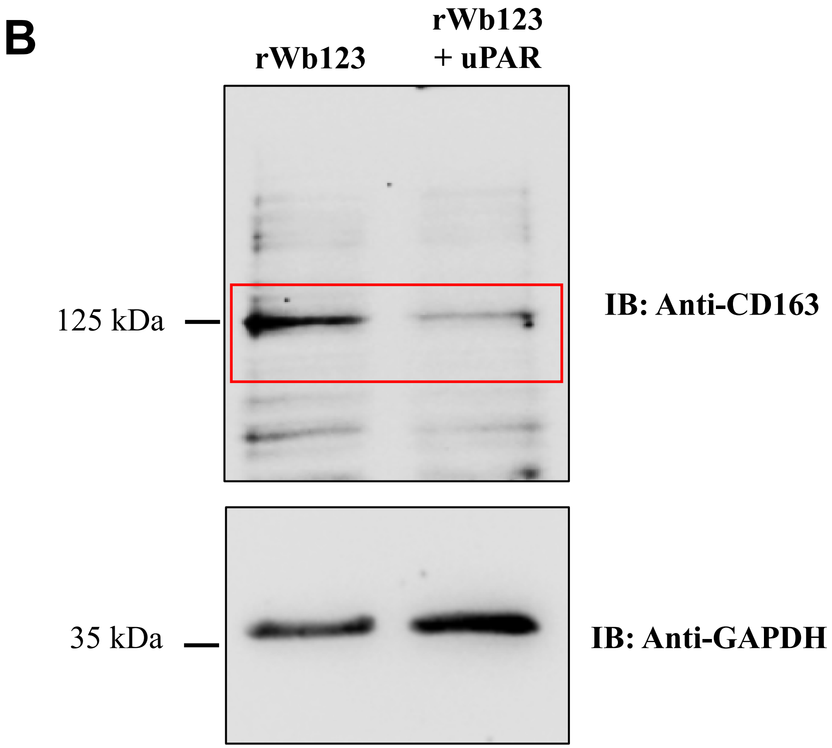

Sample	CD163_Area	GAPDH_Area	Normalised Densitometry
**rWb123**	2981980	227262	13.12133133
**rWb123+uPARAb**	904123	396097	2.282579772

A

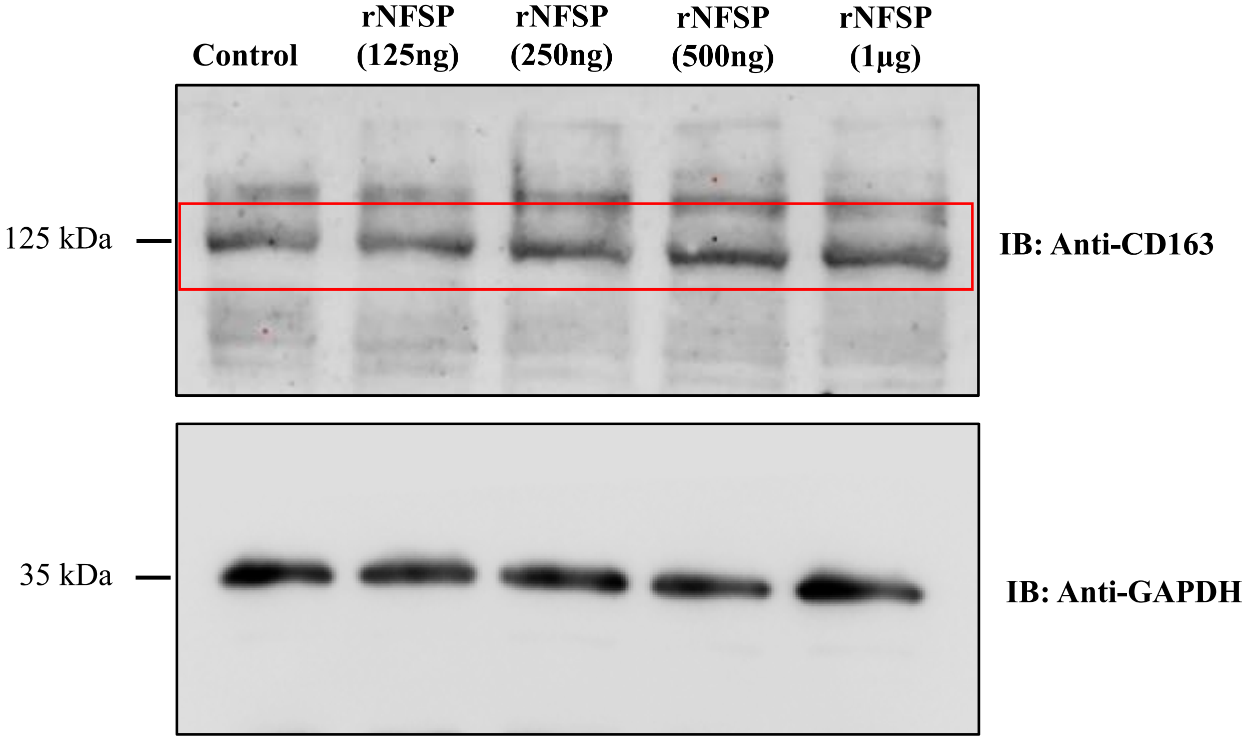

rNSFP	CD163_Area	GAPDH_Area	Normalised Densitometry
**125ng**	2438879	16241134	0.150166793
**250ng**	2270650	15414224	0.147308745
**500ng**	2825175	17207472	0.164183036
**750ng**	2926531	14617010	0.200214066
**1µg**	3083910	18323578	0.168302828

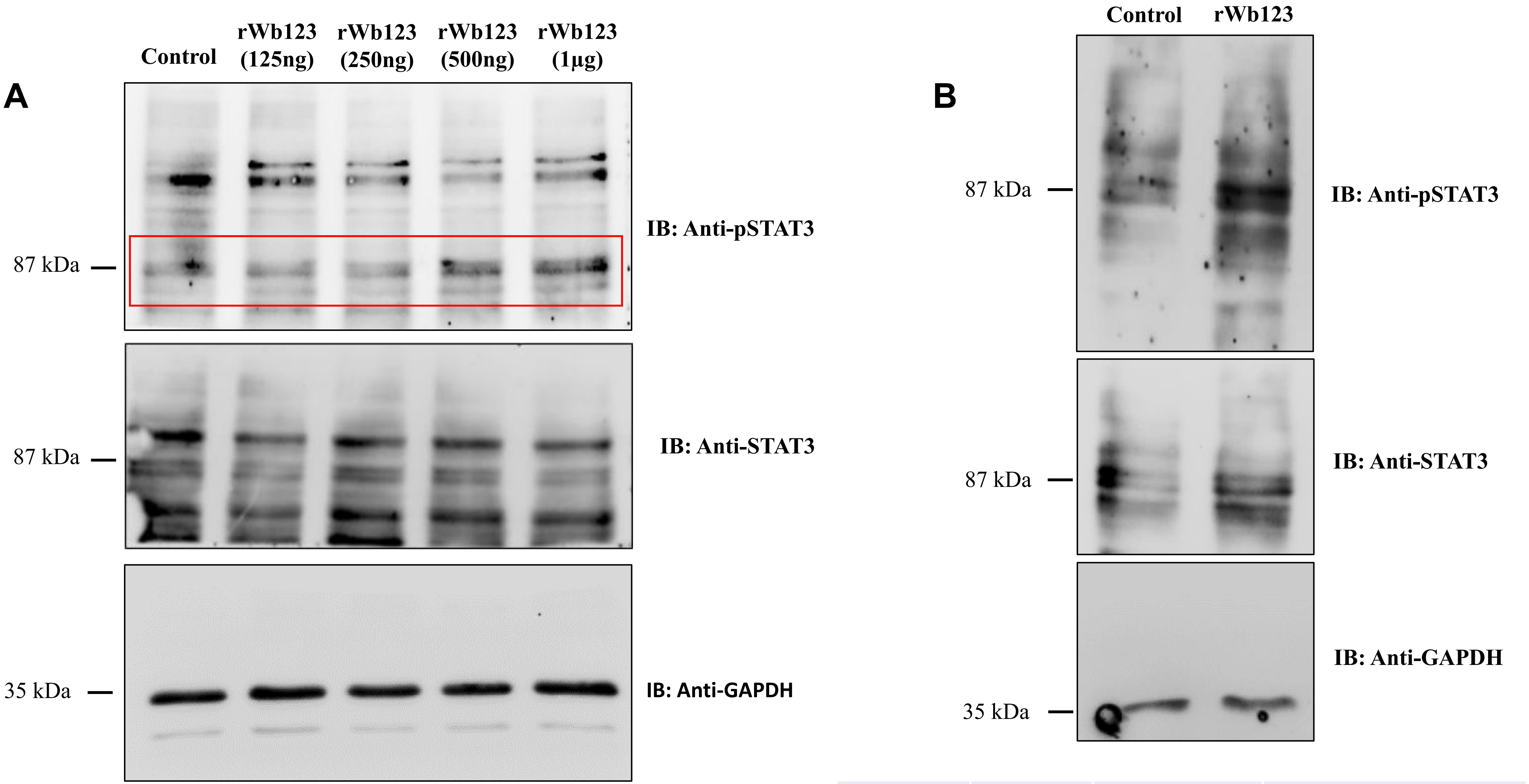

Sample	pSTAT3_Area	GAPDH_Area	Normalised Densitometry
Control	6207556	7569209	0.820106302
rWb123	12675822	11840944	1.070507723

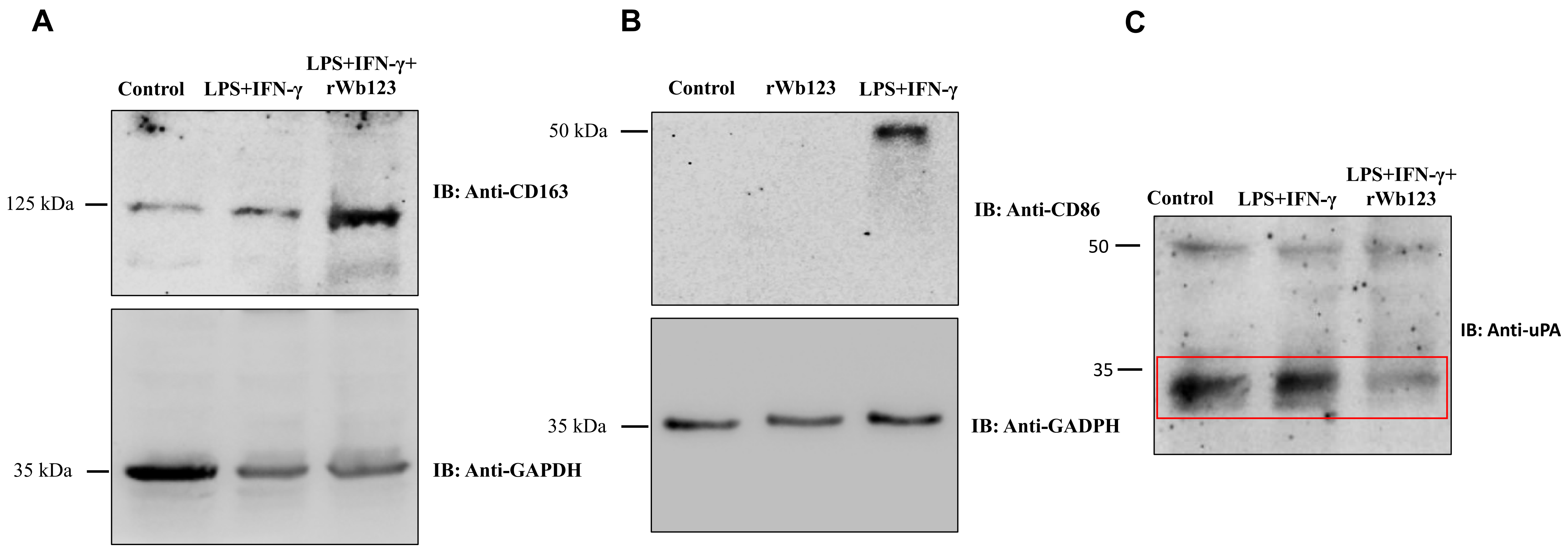

Sample	CD163_Area	GAPDH_Area	Normalised Densitometry
Control	17,77,997	52,64,821	0.337712716
LPS+IFN-γ	21,72,375	16,94,449	1.28205393
LPS+IFN-γ+rWb123	51,96,692	24,14,276	2.152484637

Sample	uPA_Area	GAPDH_Area	Normalised Densitometry
Control	13274547	4672621	2.840920973
LPS+IFN-γ	10899690	1570267	6.941297244
LPS+IFN-γ+rWb123	4185492	2453284	1.706077242

A

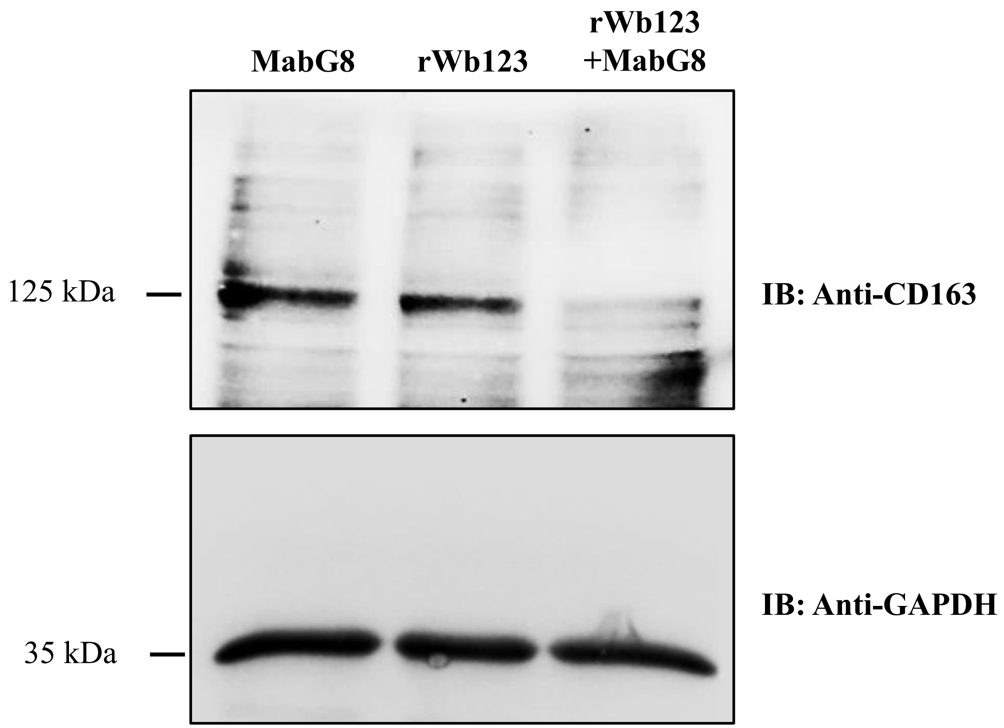

B

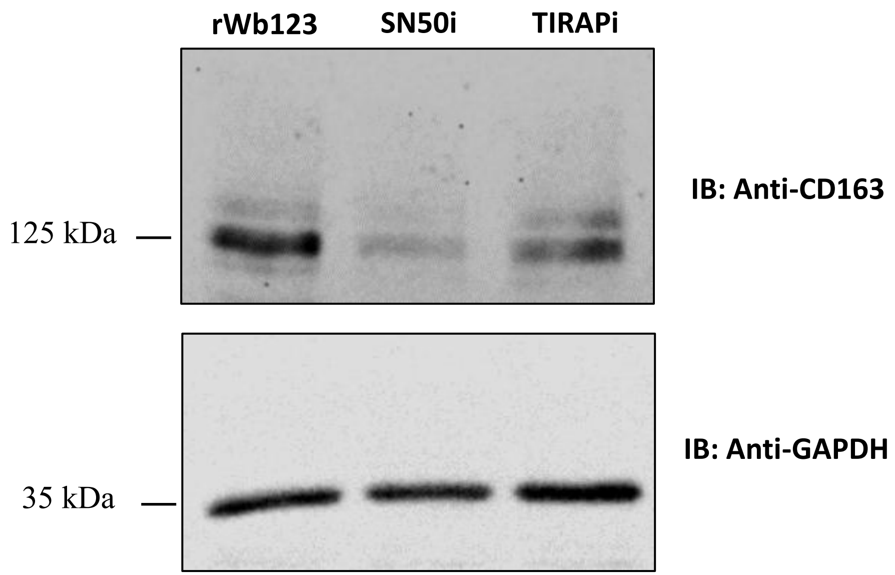

Sample	CD163_Area	GAPDH_Area	Normalised Densitometry
**Control**	1444664	6480090	0.222938879
**rWb123**	2575268	4774513	0.539378152
**rWb123+MabG8**	1046841	5089461	0.205687989
